# Evaluation of carcinogenic potential of the herbicide glyphosate, drawing on tumor incidence data from fourteen chronic/carcinogenicity rodent studies

**DOI:** 10.3109/10408444.2014.1003423

**Published:** 2015-02-26

**Authors:** Helmut Greim, David Saltmiras, Volker Mostert, Christian Strupp

**Affiliations:** ^a^ Technical University Munich, Arcisstr. 21, 80333Munich, Germany; ^b^ Monsanto Company, 800 North Lindbergh Blvd., 63167St. Louis, MO, USA; ^c^ ADAMA MAH BV Amsterdam NL Schaffhausen Branch, Spitalstrasse 5, 8200Schaffhausen, Switzerland; ^d^ Knoell Consult GmbH, Dynamostr. 19, 68165Mannheim, Germany; ^e^ Extera, Nelly-Sachs-Str. 37, 40764Langenfeld, Germany; ^f^ Glyphosate Task Force, http://www.glyphosatetaskforce.org/

**Keywords:** amino acid, carcinogenicity, epidemiology, glyphosate, herbicide, mouse, neoplasm, phosphonomethylglycine, Roundup, rat, regulatory, tumor

## Abstract

Glyphosate, an herbicidal derivative of the amino acid glycine, was introduced to agriculture in the 1970s. Glyphosate targets and blocks a plant metabolic pathway not found in animals, the shikimate pathway, required for the synthesis of aromatic amino acids in plants. After almost forty years of commercial use, and multiple regulatory approvals including toxicology evaluations, literature reviews, and numerous human health risk assessments, the clear and consistent conclusions are that glyphosate is of low toxicological concern, and no concerns exist with respect to glyphosate use and cancer in humans. This manuscript discusses the basis for these conclusions. Most toxicological studies informing regulatory evaluations are of commercial interest and are proprietary in nature. Given the widespread attention to this molecule, the authors gained access to carcinogenicity data submitted to regulatory agencies and present overviews of each study, followed by a weight of evidence evaluation of tumor incidence data. Fourteen carcinogenicity studies (nine rat and five mouse) are evaluated for their individual reliability, and select neoplasms are identified for further evaluation across the data base. The original tumor incidence data from study reports are presented in the online data supplement. There was no evidence of a carcinogenic effect related to glyphosate treatment. The lack of a plausible mechanism, along with published epidemiology studies, which fail to demonstrate clear, statistically significant, unbiased and non-confounded associations between glyphosate and cancer of any single etiology, and a compelling weight of evidence, support the conclusion that glyphosate does not present concern with respect to carcinogenic potential in humans.

## Introduction

**Figure 1.**
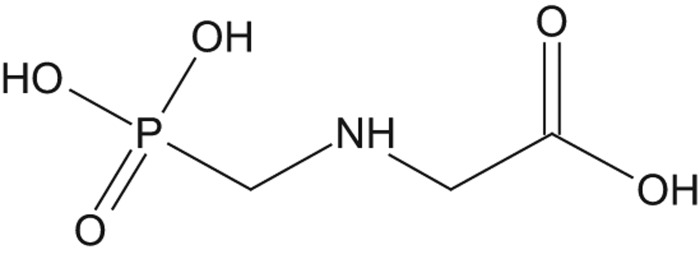
Structure of glyphosate acid.

Glyphosate (Figure 1), an aminophosphonic analog of the natural amino acid glycine, is widely used as an herbicide for the control of annual and perennial grasses and broad-leaved weeds. Glyphosate inhibits 5-enolpyruvateshikimate-3-phosphate synthase (EPSPS), an enzyme of the aromatic acid biosynthesis pathway, which is not present in the animal kingdom. Glyphosate-based herbicide formulations (GBFs) were introduced in 1974 and are formulated with sodium-, potassium-, ammonium- and isopropyl ammonium-salt forms of the active ingredient. The bulk-manufactured active herbicide glyphosate has the synonyms glyphosate technical acid, technical grade glyphosate and glyphosate acid.

The economic importance of glyphosate for growers is high. It has been estimated that a hypothetical ban of glyphosate would lead to decreases in the production of wheat, fodder, maize and oilseeds, by 4.3–7.1%, with the result of an estimated annual welfare loss of 1.4 billion USD to society in the European Union alone (Schmitz and Harvert 2012). Furthermore, glyphosate plays an important role in integrated pest management strategies, and affords the environmental benefit of substantially reduced soil erosion resulting from of no-till and reduced-till agriculture.

The long-term toxicity and carcinogenicity of glyphosate has been investigated by multiple entities including academia, registrants, and regulatory authorities, and the data generated have been evaluated in support of herbicide regulatory approvals in many world regions including the USA (US EPA 1993) and the European Union (EC 2002), and several scheduled reevaluations are currently ongoing in the USA, Canada, Japan and Europe (Germany Rapporteur Member State 2015a), with imminent conclusions.

Studies of appropriate scientific quality are the basis for regulatory decision making. Mandatory testing guidelines (TGs) exist for toxicological studies submitted for regulatory review of active substances for plant protection in many regions of the world. Such TGs have been released, *inter alia*, by the United States Environmental Protection Agency (US EPA 2012), the European Union (EU 2008), the Japanese Ministry of Agriculture, Forestry and Fisheries (JMAFF 2000), and the Organization of Economic Co-operation and Development (OECD 2012b). These TGs set quality standards for each type of study by giving guidance regarding test species, strains, and number of animals to be used, the choice of dosing, exposure duration, and parameters to be measured and observed, as well as for the reporting of results. Due to the lack of effective legal and regulatory provisions for the sharing of vertebrate study data in the past, and to guarantee the safety of technical glyphosate obtained from different processes of synthesis, several manufacturers of glyphosate had to initiate toxicological testing programs of their own. Occasionally, regulatory studies had to be repeated to reflect major changes in the underlying TG. In the case of glyphosate, this has given rise to a multitude of studies for the same toxicological endpoints, leading to the availability of an extraordinarily robust scientific study database that can be considered unique among pesticides, industrial chemicals, and pharmaceuticals. Such a remarkable volume of studies addressing the same endpoints, conducted over the last 40 years by several independent companies and laboratories while toxicology test guidelines have evolved, warrants investigation for consistency, reliability, and application to their intended purpose: identifying potential human health hazards and setting appropriate endpoints for human health risk assessment. Studies conducted with equivalent test substances using the same TG are readily comparable and can be evaluated by regulators following standardized schemes. Minor differences in the findings reported by such repetitive studies are attributable to statistical chance, natural biological variability, type of basal diet, rate of feed consumption, animal strain differences, choice of dose levels, inter-strain genetic drift over time due to varying vendor breeding practices, changes in animal care and husbandry practices across laboratories over the years, inter-laboratory variations in clinical measurements, and differences between individual pathologist evaluation and interpretation of tissue specimens.

Glyphosate is under significant political pressure due to its widespread use, particularly in association with use on genetically modified crops. One focus area of contention has been the human safety of glyphosate, which has been repeatedly challenged by interest groups via the media, as well as select research publications in the scientific literature (Antoniou et al. 2012, Aris and Leblanc 2011, Aris and Paris 2010, Benachour and Seralini 2009, Gasnier et al. 2010, Paganelli et al. 2010, Romano et al. 2012, Romano et al. 2010). To that end, one specific publication by Seralini et al. (2012, retracted) drew significant criticism from both the toxicology and broader scientific communities (Barale-Thomas 2013, Berry 2013, de Souza and Oda 2013, Grunewald and Bury 2013, Hammond et al. 2013, Langridge 2013, Le Tien and Le Huy 2013, Ollivier 2013, Panchin 2013, Sanders et al. 2013, Schorsch 2013, Tester 2013, Trewavas 2013, Tribe 2013). After a special review of the investigators’ raw data by a mutually agreed-upon expert panel, the manuscript was retracted by *Food and Chemical Toxicology* (FCT), for reasons of inconclusive data and unreliable conclusions (Hayes 2014). The Editor of the *International Journal of Toxicology* highlighted this manuscript as an example of possible failure of the peer review process in a well-respected toxicology journal with an editorial board of well-known and respected toxicologists (Brock 2014). The manuscript was later republished without peer-review in an open access journal (Seralini et al. 2014), but will not be addressed in this data evaluation due to the inappropriate study design, insufficient reporting of tumor incidence data, and the lack of a data supplementary to the manuscript.

The chronic/carcinogenicity studies discussed in this paper have been submitted to and evaluated by a variety of agencies over time, including the World Health Organization (WHO/FAO 2004b, WHO/FAO 2004a), the United States Environmental Protection Agency (US EPA 1993), the European Rapporteur Member State Germany for the initial glyphosate Annex I listing (EC 2002) and the recent European re- evaluation (Germany Rapporteur Member State 2015a), as well as the ongoing reevaluations in the USA, Canada and Japan. These regulatory bodies, drawing upon internal and/or external expertise, have consistently concluded that glyphosate is devoid of carcinogenic risk to humans.

The purpose of this article is to provide the broader scientific community with insight into this large body of carcinogenicity data on glyphosate, originally generated for regulatory purposes. Each study discussed in this review has been assigned a reliability score in Tables 3–19, following the Klimisch scoring system (Klimisch et al. 1997). In this system, a score of 1 is assigned to studies that are fully reliable based on compliance with Good Laboratory Practice (GLP) and adherence to appropriate study guidelines. A score of 2 is appropriate if some guideline requirements are not met, but if these deficiencies do not negatively affect the validity of the study for its regulatory purpose. Studies with a reliability of 3 employ a test design that is not fit for the scientific purpose of the study, due to significant scientific flaws, or the objective of the study not covering the regulatory endpoints, or both. Such studies can provide supplemental information but do not allow a stand-alone appraisal of a regulatory endpoint. No studies were assigned a reliability of 4, since each report contained sufficient information to judge the validity of the study.

**Table 1.** Summary of toxicological endpoints for glyphosate (Germany Rapporteur Member State 2015c).

Endpoint	Value	Remark
Oral absorption	*ca* 20%	Rat, *in vivo*
Dermal absorption	< 1%	Human, *in vitro*, 0.015 g glyphosate/L
Rat LD50 oral	> 2000 mg/kg bw	
Rat LD50 dermal	> 2000 mg/kg bw	
Rat LC50 inhalation	> 5 mg/L	4-h exposure
Skin irritation	Not irritating	
Eye irritation	Acid: moderately to severely irritating Salts: slight or non-irritating	
Skin sensitization	Not sensitizing (LLNA, Magnusson-Kligmant, and Buehler test)	
Genotoxicity	Not genotoxic (*in vitro* and *in vivo*)	
Chronic toxicity	BW gain, liver (organ weight ↑, clinical chemistry, histology); salivary glands (organ weight ↑, histology); stomach mucosa and bladder epithelium(histology); eye (cataracts), caecum (distention, organ weight ↑) NOAEL = 100 mg/kg bw/day (2-yr rat)	Critical study used for ADI setting
Reproductive toxicity	Reduced pup weight at parentally toxic doses. NOAEL = 300 mg/kg bw/day	
Developmental toxicity	Post-implantation loss, fetal BW & ossification ↓; effects confined to maternally toxic doses Rat NOAEL: 300 mg/kg bw/day Rabbit NOAEL: 50 mg/kg bw/day	
Delayed neurotoxicity	No relevant effects, NOAEL: 2000 mg/kg bw/day	
Acceptable Daily Intake (ADI)	0.5 mg/kg bw/day Based on developmental toxicity in rabbits	Safety factor 100
Acceptable Operator Exposure Level (AOEL)	0.1 mg/kg bw/day Based on maternal toxicity in rabbit teratogenicity study	Safety factor 100 Corrected for oral absorption of 20%

**Table 2.** Summary of one-year toxicity studies with glyphosate.

Authors:	Monsanto (1985)
Reliability/Justification	2 Study performed according to GLP and OECD guideline requirements, with the following deviation: MTD not reached by highest dose
Substance:	Glyphosate (96.1% pure)
Species/Strain:	Dog/Beagle, groups of 6 ♂ and 6 ♀
Administration route:	Oral, capsule
Doses:	0, 20, 100, 500 mg/kg bw/day
Duration:	1 year
Findings:	≥ 500 mg/kg bw/day: NOAEL (♂ + ♀) no treatment-related effects

Authors:	Cheminova (1990)
Reliability/Justification	1 Study performed according to GLP and OECD guideline requirements, with no deviations.
Substance:	Glyphosate (98.6–99.5% pure)
Species/Strain	Dog/Beagle, groups of 4 ♂ and 4 ♀
Administration route:	Oral, capsule
Doses:	0, 30, 300, 1000 mg/kg bw/day
Duration:	1 year
Findings:	300 mg/kg bw/day: NOAEL (♂ + ♀) 1000 mg/kg bw/day: soft, liquid stools (attributable to capsule administration); equivocal impact on body weight gain

Authors:	Nufarm (2007)
Reliability/Justification	2 Study performed according to GLP and OECD guideline requirements, with the following deviation: MTD not reached by highest dose
Substance:	Glyphosate (95.7% pure)
Species/Strain	Dog/Beagle, groups of 4 ♂ and 4 ♀
Administration route:	Oral, capsule
Doses:	0, 30, 125, 500 mg/kg bw/day
Duration:	1 year
Findings:	≥ 500 mg/kg bw/day: NOAEL (♂ + ♀) No treatment-related effects

Authors:	Arysta Life Sciences (1997c)
Reliability/Justification	2 Study performed according to GLP and OECD guideline requirements, with the following deviation: MTD not reached by highest dose
Substance:	Glyphosate (94.6% pure)
Species/Strain	Dog/Beagle, groups of 4 ♂ and 4 ♀
Administration route:	Oral, diet
Concentration:	0, 1600, 8000, 50 000 ppm diet (♂ about 34.1, 182, 1203 mg/kg bw/day; ♀ about 37.1, 184, 1259 mg/kg bw/day)
Duration:	1 year
Findings:	182/184 mg/kg bw/day: NOAEL (♂/♀) At high dose: loose stool, non-statistically significant retarded body weight gain, decreased urinary pH, slight and non-statistically significant focal pneumonia (♀), minor clinical chemistry changes of Cl ↑, albumin ↓, P ↓ (♀)

Authors:	Syngenta (1996a)
Reliability/Justification	1 Study performed according to GLP and OECD guideline requirements, with no deviations.
Substance:	Glyphosate (95.6% pure)
Species/Strain	Dog/Beagle, groups of 4 ♂ and 4 ♀
Administration route:	Oral, diet
Concentration:	0, 3000, 15 000, 30 000 ppm diet (♂ about 90.9, 440, 907 mg/kg bw/day; ♀ about 92.1, 448, 926 mg/kg bw/day)
Duration:	1 year
Findings:	15 000 ppm diet: NOAEL (♀) ≥ 30 000 ppm diet: NOAEL (♂): No treatment-related effects 30 000 ppm diet: slight body weight reduction (♀)

Authors:	Syngenta (1996b)
Reliability/Justification	1 Study performed according to GLP and OECD guideline requirements, with no deviations.
Substance:	Glyphosate (95.6% pure)
Species/Strain	Rat/Wistar Alpk: AP_f_SD, groups of 24 ♂ and 24 ♀
Administration route:	Oral, diet
Concentration:	0, 2000, 8000, 20 000 ppm diet (♂ about 141, 560, 1409 mg/kg bw/day; ♀ about 167, 671, 1664 mg/kg bw/day)
Duration:	1 year
Findings:	8000 ppm diet: NOAEL (♂+♀) 20 000 ppm diet: parotid salivary glands (focal basophilia of the acinar cells considered non-adverse adaptive response, ♂: 13/24, ♀: 15/24), body weight reduction

**Table 3.** Study 1–26-month feeding study of glyphosate in rats (Monsanto 1981).

Study owner:	Monsanto (1981)
Reliability/Justification:	3 Study not performed under GLP. High-dose well below MTD. Does not conform to modern testing standards.
Substance:	Glyphosate (98.7% pure)
Species/Strain:	Rat/Sprague-Dawley, groups of 50 ♂ and 50 ♀
Administration route:	Diet
Concentration:	0, 30, 100, 300 ppm diet (♂ about 0, 3, 10, 31 mg/kg bw/day; ♀ about 0, 3, 11, 34 mg/kg bw/day)
Duration:	26 months
Findings:	≥ 300 ppm diet: NOAEL (♂ + ♀) No treatment-related effects
Select neoplasms:	Pituitary adenoma, Testes interstitial cell

**Table 4.** Study 1 – Pituitary tumor findings.

Tumors	Dose group (mg/kg bw/day)							
	Males				Females			
	0	3.05	10.3	31.49	0	3.37	11.22	34.02
Pituitary tumors	Number of animals/total number examined (% per group)							
Adenomas - B	16/48 (33)	19/49 (39)	20/48 (42)	18/47 (38)	34/48 (70)	29/48 (60)	31/50 (62)	26/49 (53)
Carcinomas - M	3/48 (6)	2/49 (4)	3/48 (6)	1/47 (2)	8/48 (17)	7/48 (14)	5/48 (19)	12/49 (24)
Combined	19/48 (40)	21/49 (43)	23/48 (48)	19/47 (40)	42/48 (88)	36/48 (75)	36/50 (72)	38/49 (78)

*B* benign, *M* malignant

**Table 5.** Study 1 - Interstitial cell tumor findings in the testes.

Tumors	Dose (mg/kg bw/day)			
	0	3.05	10.3	31.49
Interstitial cell tumor – B	Number of animals/total number examined (% per group)			
Terminal sacrifice	0/15 (0)	2/26 (7.7)	1/16 (6.3)	4/26 (15.4)
All Animals	0/50 (0)	3/50 (6)	1/50 (2)	6/50 (12)
Interstitial cell hyperplasia	Number of animals (% per group)			
Terminal sacrifice	1/15 (6.7)	1/26 (3.8)	0/16 (0)	0/26 (0)
All Animals	1/50 (2)	1/50 (2)	1/50 (2)	0/50 (0)

*B* benign, *M* malignant

**Table 6.** Study 1 – Summary of the contemporary historical control data for interstitial cell tumors in the testes of rats in chronic toxicity studies.

	Study 1	Study 2	Study 3	Study 4	Study 5	Range
	Number of control animals/total number examined (% per study)					
Terminal sacrifice	4/65 (6.2)	3/11 (27.3)	3/26 (11.5)	3/24 (12.5)	3/40 (7.5)	6.2–27.3%
All animals	4/116 (3.4)	5/75 (6.7)	4/113 (3.5)	6/113 (5.3)	5/118 (4.2)	3.4–6.7%

**Table 7.** Study 2 – Two-year feeding study of glyphosate in rats (Monsanto 1990).

Study owner:	Monsanto (1990)				
Reliability/Justification:	1 Study performed according to GLP and OECD guideline requirements, with no deviations.				
Substance:	Glyphosate (96.5% pure)				
Species/Strain:	Rat/Sprague-Dawley, groups of 50 ♂ and 50 ♀ (10 rats per sex per dose were included for interim sacrifice after 12 months).				
Administration route:	Diet				
Concentration:	0, 2000, 8000, 20 000 ppm diet (♂ about 0, 89, 362, 940 mg/kg bw/day; ♀ about 0, 113, 457, 1183 mg/kg bw/day)				
Duration:	2 years				
Findings:	8000 ppm diet: NOAEL (♂+♀) 20 000 ppm diet: cataracts (♂), > 20% reduced cumulative body weight gain through months 18–20 (♀), 13% increased liver weight (♂). Local effects: inflammation of gastric mucosa				
Select neoplasms:	Pancreatic islet cell adenoma, skin keratoacanthoma (males), thyroid C cell adenoma				
Tumor			Dose (mg/kg bw/day)		
Males		0	89	362	940
Findings for dead and moribund sacrificed animals					
Pancreas: Islet call adenoma – B		1/34 (3%)	4/28 (14%)	2/33 (6%)	4/32 (13%)
Skin: Keratoacanthoma – B		0/36	1/31 (3%)	2/33 (6%)	1/32 (3%)
Thyroid: C cell adenoma – B		0/36	2/29 (7%)	1/31 (3%)	1/33 (3%)
Thyroid: C cell carcinoma – M		0/36	1/29 (3%)	2/31 (6%)	1/33 (3%)
Findings for animals sacrificed at termination					
Pancreas: Islet call adenoma – B		0/14	4/19 (21%)	3/17 (6%)	3/17 (6%)
Skin: Keratoacanthoma – B		0/13	2/19 (11%)	2/17 (12%)	2/17 (12%)
Thyroid: C cell adenoma – B		0/14	2/19 (11%)	*7/17 (41%)	4/17 (24%)
Thyroid: C cell carcinoma – M		0/14	0/19	0/17	0/17
Females		0	113	457	1183
Findings for dead and moribund sacrificed animals					
Pancreas: Islet call adenoma – B		3/28 (11%)	0/28	3/33 (9%)	0/31
Thyroid: C cell adenoma – B		0/28	0/28	1/33 (3%)	2/32 (6%)
Thyroid: C cell carcinoma – M		0/28	0/28	1/33 (3%)	0/32
Findings for animals sacrificed at termination					
Pancreas: Islet call adenoma – B		2/22 (9%)	1/22 (5%)	1/17 (6%)	0/18
Thyroid: C cell adenoma – B		2/22 (9%)	2/22 (9%)	5/17 (29%)	4/18 (22%)
Thyroid: C cell carcinoma – M		0/22	0/22	0/17	0/18

*B* benign, *M* malignant

*Statistically higher than controls (*p* < 0.05, Fisher's Exact Test with the Bonferroni Inequality).

**Table 8.** Study 3 – Two-year feeding study of glyphosate in rats (Cheminova 1993a).

Study owner:	Cheminova (1993a)
Reliability/Justification:	1 Study performed according to GLP and OECD guideline requirements, with no deviations.
Substance:	Glyphosate (98.7–98.9% pure)
Species/Strain:	Rat/Sprague-Dawley, groups of 50 ♂ and 50 ♀ (additional groups of 35 ♂ and 35 ♀per dose were included for 1-year interim sacrifice)
Administration route:	Diet
Achieved dose:	♂+♀: 0, 10, 100, 300, 1000 mg/kg bw/day (weekly adjustment of dietary concentration for the first 13 weeks and 4-weekly thereafter)
Duration:	2 years
Findings:	300 mg/kg bw/day: NOAEL (♂+♀) 1000 mg/kg bw/day: body weights ↓, urinary pH ↓, salivary glands (histopathology, organ weight ↑); evidence of weak liver toxicity (alkaline phosphatase ↑, ♀: organ weight ↓)
Select neoplasms:	No neoplasms from this study were identified for further consideration.

**Table 9.** Study 4 – Two-year feeding study of glyphosate in rats (Feinchemie Schwebda 1996).

Study owner:	Feinchemie Schwebda (1996)				
Reliability/Justification:	1 Study performed according to GLP and OECD guideline requirements, with no deviations.				
Substance:	Glyphosate (96.0–96.8% pure)				
Species/Strain:	Rat/Wistar, groups of 50 ♂ and 50 ♀				
Administration route:	Diet				
Concentration:	0, 100, 1000, 10 000 ppm diet (♂ about 0, 6.3, 59.4, 595 mg/kg bw/day; ♀ about 0, 8.6, 88.5, 886 mg/kg bw/day)				
Duration:	2 years				
Findings:	10 000 ppm diet: ≥ NOAEL (♂+♀) Only mild effects on clinical chemistry (liver enzymes), without histopathological changes.				
Select neoplasms:	Hepatocellular adenoma, hepatocellular carcinoma				
Tumor			Dose (mg/kg bw/day)		
Males		0	7.4	73.9	741
Findings for dead and moribund sacrificed animals					
Hepatocellular adenoma – B		9/30 (30%)	9/30 (30%)	6/32 (19%)	6/21 (29%)
Hepatocellular carcinoma – M		12/30 (40%)	12/30 (40%)	9/32 (28%)	5/21 (24%)
Findings for animals sacrificed at termination					
Hepatocellular adenoma – B		15/20 (75%)	13/20 (65%)	4/16 (25%)	15/20 (75%)
Hepatocellular carcinoma – M		9/20 (45%)	16/20 (80%)	9/16 (56%)	19/29 (66%)
			Dose (mg/kg bw/day)		
Females		0	7.4	73.9	741
Findings for dead and moribund sacrificed animals					
Hepatocellular adenoma – B		2/26 (8%)	8/23 (3%)	3/17 (18%)	5/29 (17%)
Hepatocellular carcinoma – M		4/26 (15%)	4/23 (17%)	2/17 (12%)	5/29 (17%)
Findings for animals sacrificed at termination					
Hepatocellular adenoma – B		16/24 (67%)	10/25 (40%)	16/32 (50%)	8/21 (38%)
Hepatocellular carcinoma – M		6/24 (25%)	11/25 (44%)	12/32 (38%)	4/21 (19%)

*B* benign, *M* malignant

**Table 10.** Study 5 – Two-year feeding study of glyphosate in rats (Excel 1997).

Study owner:	Excel (1997)			
Reliability/Justification:	3 Test substance not characterized and other deviations from OECD 453, lower than expected background tumor incidence			
Substance:	Glyphosate (no purity reported)			
Species/Strain:	Rat/Sprague-Dawley, groups of 50 ♂ and 50 ♀, additional groups of 20 rats per sex and group were included for interim sacrifice after 52 weeks			
Administration route:	Diet			
Concentration:	2-year group: 0, 3000, 15 000, 25 000 ppm diet (♂ about 0, 150, 780, 1290 mg/kg bw/day; ♀ about 0, 210, 1060, 1740 mg/kg bw/day) 1-year group: 0, 3000, 15 000, 30 000 ppm diet (♂ about 0, 180, 920, 1920 mg/kg bw/day; ♀ about 0, 240, 1130, 2540 mg/kg bw/day)			
Duration:	2 years			
Findings:	≥ 25 000 ppm diet: NOAEL (♂+♀) Only mild toxic effects, such as clinical chemistry of questionable relevance in aged rats, without correlating histopathological organ changes.			
Select neoplasms:	No neoplasms from this study were identified for further consideration. Low background tumor incidence indicates low study reliability with no relevant increases in the incidence of tumors.			
Males	Dose (mg/kg bw/day)			
	0	150	740.6	1290
Mortality	16/50 (32%)	17/50 (34%)	18/50 (36%)	23/50 (46%)
Females	Dose (mg/kg bw/day)			
	0	210	1060	1740
Mortality	19/50 (38%)	20/50 (40%)	20/50 (40%)	25/50 (50%)

**Table 11.** Study 6 – Two-year feeding study of glyphosate in rats (Arysta Life Sciences 1997b).

Study owner:	Arysta Life Sciences (1997b)					
Reliability/Justification:	1 Study performed according to GLP and OECD guideline requirements, with no deviations.					
Substance:	Glyphosate (94.6–97.6% pure)					
Species/Strain:	Rat/Sprague-Dawley, groups of 50 ♂ and 50 ♀; satellite groups of 30 ♂ and 30 ♀for interim investigations					
Administration route:	Diet					
Concentration:	0, 3000, 10 000, 30 000 ppm diet (♂ about 0, 104, 354, 1127 mg/kg bw/day; ♀ about 0, 115, 393, 1247 mg/kg bw/day)					
Duration:	2 years					
Findings:	3000 ppm diet: NOAEL (♂+♀) 10 000 ppm diet: cecum weight↑, distension of cecum, loose stool, follicular hyperkeratosis and/or folliculitis/follicular abscess of the skin, body weight ↓					
Select neoplasms:	Pituitary adenoma, skin keratoacanthoma (males), mammary gland fibroadenoma (females)					
Tumor			Dose (mg/kg bw/day)			
Males			0	104	354	1127
Findings for dead and moribund sacrificed animals (Table 25–10)						
Pituitary anterior adenoma – B			22/32 (69%)	21/30 (70%)	*14/32 (44%)	18/21 (86%)
Skin keratoacanthoma – B			2/32 (6%)	1/30 (3%)	0/32	1/21 (5%)
Findings for animals sacrificed at termination (after 104 weeks, Table 25–8)						
Lung adenoma – B			0/18	2/20 (10%)	1/18 (6%)	3/29 (10%)
Pituitary anterior adenoma – B			13/18 (72%)	14/20 (70%)	13/18 (72%)	21/29 (72%)
Pituitary adenoma in intermediate part – B			0/18	1/20 (5%)	0/18	0/29 (0%)
Skin keratoacanthoma – B			1/18 (6%)	2/20 (10%)	0/18	6/29 (21%)
Tumor				Dose (mg/kg bw/day)		
Females			0	115	393	1247
Findings for dead and moribund sacrificed animals						
Pituitary anterior adenoma – B			34/35 (97%)	29/31 (94%)	28/33 (82%)	31/36 (86%)
Thyroid follicular adenoma – B			0/35	2/31 (6%)	0/32	0/36
Mammary gland fibroadenoma – B			13/35 (37%)	14/31 (45%)	12/34 (35%)	20/36 (56%)
Findings for animals sacrificed at termination						
Pituitary anterior adenoma – B			12/15 (80%)	19/19 (100%)	12/16 (75%)	13/14 (93%)
Mammary gland fibroadenoma – B			10/15 (67%)	13/19 (68%)	12/16 (75%)	10/14 (71%)

*B* benign, *M* malignant

*Statistically lower than controls (*p* < 0.05).

**Table 12.** Study 7 – Two-year feeding study of glyphosate in rats (Syngenta 2001).

Study owner:	Syngenta (2001)			
Reliability/Justification	1 Study performed according to GLP and OECD guideline requirements, with no deviations.			
Substance:	Glyphosate (97.6% pure)			
Species/Strain	Rat/Wistar Alpk: AP_f_SD, groups of 52 ♂ and 52 ♀ (additional 12 animals per sex and dose for 1-year interim sacrifice)			
Administration route:	Diet			
Concentration:	0, 2000, 6000, 20 000 ppm diet (♂ about 0, 121, 361, 1214 mg/kg bw/day; ♀ about 0, 145, 437, 1498 mg/kg bw/day)			
Duration:	2 years			
Findings:	6000 ppm diet: NOAEL (♂+♀) 20 000 ppm diet: Kidney and liver findings. Increased survival due to reduction in CPN, prostatitis, periodontal inflammation			
Select neoplasms:	Hepatocellular adenoma (males), not a statistically significant increase for the high dose using the Fisher's exact test, but statistically significant using Peto trend analysis			
		Dose (mg/kg bw/day)		
Males	0	121	361	1214
*Liver*				
Hepatocyte fat vacuolation	6	7	11	11
Hepatitis	3	4	2	5
*Kidney*				
	Dose (mg/kg bw/day)			
Females	0	145	437	1498
*Liver*				
Hepatocyte fat vacuolation	7	5	6	6
Hepatitis	6	5	4	4
Tumors:	Dose (mg/kg bw/day)			
Males	0	121	361	1214
Findings for dead and moribund sacrificed animals				
*Hepatocellular adenoma – B	0/37	2/36 (6%)	0/35	3/26 (12%)
Hepatocellular carcinoma – M	0/37	0/36	0/35	0/26
Findings for animals sacrificed at termination				
*Hepatocellular adenoma – B	0/16	0/17	0/18	2/26 (8%)
Hepatocellular carcinoma – M	0/16	0/17	0/18	0/26

*B* benign, *M* malignant

*Historical Control Range: 0–11.5% total males with hepatocellular adenoma, 26 studies, 1984–2003

**Table 13.** Study 8 – Two-year feeding study of glyphosate in rats (Nufarm 2009b).

Study owner:	Nufarm (2009a)				
Reliability/Justification:	1 Study performed according to GLP and OECD guideline requirements, with no deviations				
Substance:	Glyphosate (95.7% pure)				
Species/Strain:	Rat/Wistar, groups of 51 ♂ and 51 ♀				
Administration route:	Diet				
Concentration:	0, 3000, 10 000, 15 000 ppm diet, the top dose was progressively increased to reach 24 000 ppm diet by Week-40 (♂ about 0, 84, 285, 1077 mg/kg bw/day; ♀ about 0, 105, 349, 1382 mg/kg bw/day)				
Duration:	2 years				
Findings:	≥ 1077/1382 mg/kg bw/day: NOAEL (♂/♀) Transient liver enzyme activity for mid-dose males and high-dose males and females; equivocal nephrocalcinosis depositions at the high-dose males and females; increased adipose infiltration of the bone marrow in high-dose males				
Select neoplasms:	Skin keratoacanthoma (males), mammary gland adenocarcinoma				

Tumor		Dose (mg/kg bw/day)			
Males		0	84	285	1077
Findings for all animals					
Skin keratoacanthoma – B		2/51 (4%)	3/51 (6%)	0/51	6/51 (12%)
		Dose (mg/kg bw/day)			
Females		0	105	349	1382
Findings for all animals					
Mammary gland adenocarcinoma – M		2/51 (4%)	3/51 (6%)	1/51 (2%)	6/51 (12%)

*B* benign, *M* malignant

**Table 14.** Publication, Study 9 – Two-year drinking water study in rats with 13.85% glyphosate ammonium salt (Chruscielska et al. 2000a).

Authors:	Chruscielska et al. (2000a)								
Reliability/Justification:	3 Study not performed according to GLP, but according to OECD TG 453, with the following deficiencies: Reporting deficits (water and feed consumption, body weights, diet composition, individual animal data, substance composition, purity, and stability) Highest dose did not elicit toxicity.								
Substance:	Ammonium salt of glyphosate, 13.85% solution								
Species/Strain:	Rat/Wistar -RIZ outbred, 85 ♂ and 85 ♀ per dose group. 10 ♂ and 10 ♀each were sacrificed after 6, 12, and 18 months of exposure.								
Administration route:	Drinking water								
Concentration:	0, 300, 900, and 2700 mg/L Estimated glyphosate intake: ♂: 0, 1.9, 5.7, and 17 mg/kg bw/day. ♀: 0, 2.2, 6.5, and 19 mg/kg bw/day, based on assumed water consumptions of 50/57 mL/kg bw/day (♂/♀), (Gold, et al. 1984)								
Duration:	2 years								
Findings:	17/19 mg glyphosate/kg bw/day: NOAEL (♂/♀) No treatment-related effects								
Tumors reported for 85 rats/sex/dose:	No increase in the incidence of tumors attributable to glyphosate administration								
	Estimated dose (mg/kg bw/day)								
		0		1.9/2.2		5.7/6.5		17/19	
		♂	♀	♂	♀	♂	♀	♂	♀
Two-year mortality		42%	38%	42%	45%	54%	53%	44%	60%
Lungs									
Lymphoma		2	–	2	–	1	–	3	1
Histiocytoma		–	–	–	–	–	–	–	1
Adenocarcinoma		1	–	–	–	–	–	–	–
Histiocytoma, malignant		–	1	–	–	1	–	–	–
Spleen, leukemia		0	–	2	–	0	–	1	–
Kidneys, Fibrous histiocytoma		–	–	–	–	–	–	1	–
Pituitary gland									
Adenoma		4	10	4	6	2	8	0	3
Adenoma, malignant (assumed to be carcinoma)		0	1	0	3	1	2	1	5
Carcinoma		0	–	0	–	1	–	0	–
Thyroid									
Adenoma		1	1	1	2	0	0	3	3
Carcinoma		0	–	1	–	0	–	0	–
Uterus, cervix carcinoma		–	0	–	0	–	0	–	1
Uterus, body, histiocytoma		–	3	–	1	–	0	–	1
Mammary gland									
Fibroma		–	0	–	0	–	0	–	0
Fibroadenoma		–	3	–	2	–	3	–	3
Adrenal medulla, adenoma		1	2	2	2	1	2	0	2
Thymus, lymphoma		0		0		0		1	
Testis, Leydigoma		–		3		6		1	
Subcutaneous tissue									
Fibroma		0		1		1		3	
Lipoma		–	–	–	–	–	–	–	1
Cystadenoma		–	1	–	–	–	–	–	–
Lymph nodes									
Lymphoma		0		0		0		1	
Lymphoma, malignant		–	1	–	–	–	–	–	–
Skin, carcinoma		2	–	–	–	–	–	–	–
Prostate, adenoma		1	–	–	–	–	–	–	–

**Table 15.** Study 10 – Two-year feeding study with glyphosate in mice (Monsanto 1983).

Study owner:	Monsanto (1983)				
Reliability/Justification	2 Study was performed prior to institution of GLP and OECD guideline requirements				
Substance:	Glyphosate (99.7% pure)				
Species/Strain:	Mouse/CD-1, groups of 50 ♂ and 50 ♀				
Administration route:	Diet				
Concentration:	0, 1000, 5000, 10 000 ppm diet (♂ about 0, 157, 814, 4841 mg/kg bw/day; ♀ about 0, 190, 955, 5874 mg/kg bw/day)				
Duration:	24 months				
Findings:	1000 ppm diet: NOAEL (♂ + ♀) 5000 ppm diet: body weight ↓, histological changes in liver and urinary bladder (slight to mild epithelial hyperplasia in males at mid and high doses)				
Select neoplasms:	Lymphoreticular neoplasms, bronchiolar-alveolar adenocarcinoma				
			Dose (mg/kg bw/day)		
Males		0	157	814	4841
Lymphoreticular system					
Lymphoblastic lymphosarcoma with leukemia – M		1/48 (2%)	4/49 (8%)	3/50 (6%)	2/49 (4%)
Lymphoblastic lymphosarcoma without leukemia – M		0/48	1/49 (2%)	0/50 (0%)	0/49
Composite lymphosarcoma – M		1/48 (2%)	0/49	1/50 (2%)	0/49
Histiocytic sarcoma – M		0/48	1/49 (2%)	0/50	0/49
Total lymphoreticular neoplasms^#^		2/48 (4%)	6/49 (12%)	4/50 (8%)	2/49 (4%)
			Dose (mg/kg bw/day)		
Females		0	190	955	5873
Lymphoreticular system					
Lymphoblastic lymphosarcoma with leukemia – M		1/50 (2%)	4/48 (8%)	5/49 (10%)	1/49 (2%)
Lymphoblastic lymphosarcoma without leukemia – M		0/50 (0%)	1/48 (2%)	0/49 (0%)	3/49 (6%)
Composite lymphosarcoma – M		4/50 (8%)	1/48 (2%)	1/49 (2%)	6/49 (12%)
Histiocytic sarcoma – M		0/50 (0%)	0/48 (0%)	0/49 (0%)	0/49 (0%)
^#^ Total lymphoreticular neoplasms		5/50 (10%)	6/48 (13%)	6/49 (12%)	10/49 (20%)

^#^Sum of lymphoblastic lymphosarcoma, composite lymphosarcoma, and histiocytic sarcoma.

*M* malignant

**Table 16.** Study 11 – Two-year feeding study with glyphosate in mice (Cheminova 1993b).

Study owner:	Cheminova (1993b)				
Reliability/Justification:	1 Study performed according to GLP and OECD guideline requirements				
Substance:	Glyphosate (98.6% pure)				
Species/Strain:	Mouse/CD-1, groups of 50 ♂ and 50 ♀				
Administration route:	Diet				
Concentration:	♂+♀: 0, 100, 300, 1000 mg/kg bw/day (regular adjustment of dietary concentration)				
Duration:	24 months				
Findings:	≥ 1000 mg/kg bw/day: NOAEL (♂+♀) no treatment-related effects				
Select neoplasms:	Bronchiolar-alveolar adenoma, bronchiolar-alveolar carcinoma, pituitary adenoma (females)				
			Dose (mg/kg bw/day)		
Males		0	10	300	1000
Bronchiolar-alveolar adenoma – B		9/50 (18%)	15/50 (30%)	11/50 (22%)	13/50 (26%)
Bronchiolar-alveolar carcinoma – M		10/50 (20%)	7/50 (14%)	8/50 (16%)	9/50 (18%)
			Dose (mg/kg bw/day)		
Females		0	100	300	1000
Bronchiolar-alveolar adenoma – B		7/50 (14%)	3/50 (6%)	3/50 (6%)	6/50 (12%)
Bronchiolar-alveolar carcinoma – M		3/50 (6%)	2/50 (4%)	1/50 (2%)	5/50 (10%)
Pituitary adenoma – B		1/41 (2%)	0/32	0/23	3/43 (6%)

*B* benign, *M* malignant

**Table 17.** Study 12 – Two-year feeding study with glyphosate in mice (Arysta Life Sciences 1997a).

Study owner:	Arysta Life Sciences (1997b)				
Reliability/Justification:	1 Study performed according to GLP and OECD guideline requirements, with no deviations.				
Substance:	Glyphosate (94.6–97.6% pure)				
Species/Strain	Mouse/CD-1, groups of 50 ♂ and 50 ♀				
Administration route:	Diet				
Concentration:	0, 1600, 8000, or 40 000 ppm diet (♂ about 0, 165, 838, 4348 mg/kg bw/day; ♀ about 0, 153, 787, 4116 mg/kg bw/day)				
Duration:	18 months				
Findings:	8000/1600 ppm diet: NOAEL (♂/♀) 8000 ppm diet (♀): retarded growth 40 000 ppm diet: pale-colored skin ♂, loose stool, retarded growth, reduced food consumption and food efficiency, cecum distension and increased absolute and relative cecum weight, without histopathological findings of increased incidence of anal prolapse, consistent with histopathological erosion/ulcer of the anus				
Select neoplasms:	Lung adenoma, lung adenocarcinoma, lymphoma				
			Dose (mg/kg bw/day)		
Males		0	165	838	4348
Lung adenoma – B		8/50 (16%)	14/50 (28%)	13/50 (26%)	11/50 (11%)
Lung adenocarcinoma – M		1/50 (2%)	1/50 (2%)	6/50 (12%)	4/50 (8%)
Lymphoma – M		2/50 (4%)	2/50 (4%)	0/50	6/50 (12%)
			Dose (mg/kg bw/day)		
Females		0	153	787	4116
Lung adenoma – B		8/50 (16%)	5/50 (10%)	12/50 (24%)	5/50 (10%)
Lung adenocarcinoma – M		1/50 (2%)	2/50 (4%)	3/50 (6%)	1/50 (2%)
Lymphoma – M		6/50 (12%)	4/50 (8%)	8/50 (16%)	7/50 (14%)

*B* benign, *M* malignant

**Table 18.** Study 13–18-Month feeding study with glyphosate in mice (Feinchemie Schwebda 2001).

Study owner:	Feinchemie Schwebda (2001)					
Reliability/Justification	2 Study performed according to GLP and OECD guideline requirements, with no deviations, but possible viral infection may have confounded interpretation of results					
Substance:	Glyphosate (> 95% pure)					
Species/Strain	Mouse/Swiss albino, groups of 50 ♂ and 50 ♀					
Administration route:	Diet					
Concentration:	0, 100, 1000, 10 000 ppm diet (♂ about 0, 14.5, 150, 1454 mg/kg bw/day; ♀ about 0, 15.0, 151, 1467 mg/kg bw/day)					
Duration:	18 months					
Findings:	1000 ppm diet: NOAEL (♂+♀) 10 000 ppm diet (♂+♀): increased mortality					
Select neoplasms:	Bronchiolar/alveolar adenoma, lymphoma					
	Historical controls			Dose (mg/kg bw/day)		
			0	14.5	150	1454
Males						
Mortality	^§^11/50–27/50		^+ ^22/50 (6)	20/50 (6)	22/50 (8)	27/50 (8)
Findings for dead and moribund sacrificed animals						
Lymphoma – M	^#^20/75	26.7% [0–44]	9/22 (41.0%)	*12/20 (60.0%)	*13/22 (59.0%)	13/27 (48.0%)
Findings in animals sacrificed at termination						
Lymphoma – M	26/175	14.9% [8–24]	1/28 (3.6%)	3/30 (10.0%)	3/28 (10.7%)	*6/23 (26.1%)
Total animals						
Lymphoma – M	46/250	18.4% [6–30]	10/50 (20.0%)	15/50 (30.0%)	16/50 (32.0%)	*19/50 (38.0%)
	Historical controls			Dose (mg/kg bw/day)		
			0	15.0	151	1467
Females						
Mortality	12/50–20/50		16/50 (7)	16/50 (7)	20/50 (2)	20/50 (3)
Findings for dead and moribund sacrificed animals						
Bronchiolar/alveolar adenoma – B	−	−	0/16	0/16	1/20 (5%)	2/20 (10%)
Lymphoma – M	49/77	63.6% [0–100]	9/16 (56.0%)	10/16 (63.0%)	13/20 (65.0%)	12/20 (60.0%)
Findings in animals sacrificed at termination						
Bronchiolar/alveolar adenoma – B			1/34 (3%)	0/0	1/1 (100%)	1/30 (3%)
Lymphoma – M	50/175	28.9% [2043]	9/34 (26.5%)	10/30 (29.4%)	6/30 (20.0%)	*13/28 (43.3%)
Total animals						
Bronchiolar/alveolar adenoma – B			1/50 (2%)	0/16	2/21 (10%)	3/50 (6%)
Lymphoma – M	99/250	39.6% [1458]	18/50 (36.0%)	20/50 (40.0%)	19/50 (38.0%)	*25/50 (50.0%)

*B* benign, *M* malignant.

^§^Nine studies, performed by the same laboratory in the timeframe encompassing the study summarized here.

^+^(Number of animals killed in extremis).

^#^Five studies, conducted in the same laboratory between 1996 and 1999.

*Statistically higher than concurrent controls (*p* < 0.05).

**Table 19.** Study 14–18-Month feeding study with glyphosate in mice (Nufarm 2009a).

Study owner:	Nufarm (2009b)			
Reliability/Justification:	1 Study performed according to GLP and OECD guideline requirements, with no deviations			
Substance:	Glyphosate (94.6–97.6% pure)			
Species/Strain:	mouse/CD-1, groups of 51 ♂ and 51 ♀			
Administration route:	Diet			
Concentration:	0, 500, 1500, and 5000 ppm diet (♂ about 0, 0, 71.4, 234, 810 mg/kg bw/day; ♀ about 0, 97.9, 300, 1081 mg/kg bw/day)			
Duration:	18 months			
Findings:	≥ 5000 ppm diet: NOAEL (♂/♀) No treatment-related effects			
Select neoplasms:	Bronchiolar-alveolar adenoma, Bronchiolar-alveolar adenocarcinoma, hepatocellular adenoma (males), hepatocellular carcinoma (males), lymphoma, pituitary adenoma (females)			
		Dose (mg/kg bw/day)		
Males	0	157	814	4841
Bronchiolar-alveolar adenoma – B	9/51 (18%)	7/51 (14%)	9/51 (18%)	4/51 (8%)
Bronchiolar-alveolar adenocarcinoma – M	5/51 (10%)	5/51 (10%)	7/51 (14%)	11/51 (22%)
Hepatocellular adenoma – B	1/51 (2%)	1/51 (2%)	4/51 (8%)	2/51 (4%)
Hepatocellular carcinoma – M	6/51 (12%)	11/51 (22%)	7/51 (14%)	4/51 (8%)
Lymphoma – M	0/51	1/50 (2%)	2/51 (4%)	5/51 (10%)
		Dose (mg/kg bw/day)		
Females	0	190	955	5873
Bronchiolar-alveolar adenoma – B	2/51 (4%)	4/51 (8%)	2/51 (4%)	2/51 (4%)
Bronchiolar-alveolar adenocarcinoma – M	5/51 (10%)	2/51 (4%)	2/51 (4%)	3/51 (6%)
Lymphoma – M	11/51 (22%)	8/51 (16)	10/51 (20%)	11/51 (22%)
Pituitary adenoma – B	0/51	1/50 (2%)	0/51	2/51 (4%)

*B* benign, *M* malignant

This manuscript presents the robust glyphosate carcinogenicity data generated by industry. Study summaries will focus on carcinogenicity evaluation, to allow third parties the opportunity to independently evaluate the carcinogenicity data presented alongside other relevant data on carcinogenicity, i.e. genotoxicity testing and epidemiology, and facilitate a multidisciplinary carcinogenicity assessment as proposed in the literature, by recognized experts in the fields of toxicology and human health risk assessment (Adami et al. 2011).

## Absorption, distribution, metabolism and excretion of glyphosate

A number of absorption, distribution, metabolism, and excretion studies (ADME) have been conducted on glyphosate for evaluation in regulatory submissions (EC 2002, US EPA 1993, WHO/FAO 2004a) and also by academic institutions (Anadon et al. 2009). Glyphosate consistently demonstrates low gastrointestinal absorption (20–40%). Its metabolism is very limited, whereby only small quantities of a single metabolite, aminomethylphosphonic acid (AMPA), are eliminated in feces. AMPA is likely produced by the limited metabolism of glyphosate by the gastrointestinal microflora, rather than via mammalian metabolism. Glyphosate is structurally akin to a phase II metabolite, a glycine-conjugate of methyl phosphonate, and thus avails itself to rapid urinary excretion. Systemic elimination is biphasic, with alpha-phase half-lives in the range of 6–14 h (Anadon et al. 2009, WHO/FAO 2004a).

## Toxicological properties of glyphosate

Table 1 contains a short overview of toxicological endpoints of glyphosate that have been published in the List of Endpoints identified for glyphosate by the Rapporteur in the European Union under Regulation 1107/2009 (Germany Rapporteur Member State 2015c). Glyphosate is of low acute toxicity via all routes of exposure. Glyphosate's active ingredient, an organic acid, has an irritating effect on mucosa which is evidenced by eye irritation and effects on oral and gastrointestinal mucosa; final formulated products contain more neutral pH salt forms, as reflected in the tabulated eye irritation data reported in Table 11, on page 109 of the 2004 JMPR Toxicological Evaluation (WHO/FAO 2004a). Glyphosate is not mutagenic, not neurotoxic, and has no effect on pre-natal development and fertility at doses not exceeding the maximum tolerated dose (MTD).

## Genotoxicity

Very recently, a review of the vast body of genotoxicity studies on glyphosate and GBFs has been published (Kier and Kirkland 2013), including an online data supplement presenting detailed data from 66 separate *in vitro* and *in vivo* genotoxicity assays. The authors incorporated these studies and published genotoxicity data into a weight-of-evidence analysis. The vast majority (over 98%) of the available bacterial reversion and *in vivo* mammalian micronucleus and chromosomal aberration assays were negative. Negative results for *in vitro* gene mutation and a large majority of negative results for clastogenic effect assays in mammalian cells support the conclusion that glyphosate is not genotoxic for these endpoints in mammalian test systems. DNA damage effects are reported in some instances for glyphosate at high or toxic dose levels. The compelling weight of evidence is that glyphosate and typical GBFs are negative in core assays, indicating that the reported high-dose effects are secondary to toxicity and are not due to DNA-reactive mechanisms. Mixed results were observed for micronucleus assays in non-mammalian systems and DNA damage assays of GBFs. These effects of GBFs may also be associated with surfactants present in the formulated products. Kier and Kirkland conclude that glyphosate and its typical formulations do not present significant genotoxic risk under normal conditions of human or environmental exposures.

## Epidemiology

Available epidemiological studies of glyphosate and cancer endpoints were recently reviewed (Mink et al. 2012). Seven cohort studies and fourteen case-control studies examining a potential association between glyphosate and one or more cancer outcomes were subjected to a qualitative analysis. The review found no consistent pattern of positive associations between total cancer (in adults or children) or any site-specific cancer, and exposure to glyphosate. A recent review article (Alavanja et al. 2013) cites one epidemiology study associating glyphosate use with non-Hodgkin's lymphoma (NHL), and accepts the study findings *prima facie*. However, Alavanja et al. (2013) did not highlight six other published epidemiology studies which evaluated glyphosate use and NHL, noting that any association between NHL and glyphosate use was null or not statistically significant. All seven studies were scrutinized by Mink et al. (2012). NHL is not a specific disease, as mentioned in both the epidemiology review publications above, but is rather multiple presentations of lymphoma which are simplistically classified as not being Hodgkin's lymphoma (HL). This dichotomous classification of HL/NHL was rejected by the World Health Organization in 2001, whereby 43 different lymphomas of various etiologies were precisely characterized (Berry 2010). The Bradford Hill criteria are often applied in efforts to determine whether an association between a health effect and human exposure may be deemed causal. However, an important premise often overlooked from Sir Austin Bradford Hill's famous speech of 1965, is that before applying these criteria, the observations should “reveal an association between two variables, perfectly clear-cut and beyond what we care to attribute to the play of chance” (Bradford Hill 1965). This predicate of the association being “perfectly clear-cut” was recently highlighted as requiring statistical significance, wherein the confidence interval of a relative risk ratio is bracketed above 1.0, as well as concluding that the association may not be attributable to bias, confounding or sampling error (Woodside and Davis 2013). According to Bradford Hill, should an epidemiology study be considered to demonstrate a “perfectly clear-cut” association between glyphosate exposure and a human health outcome, only then should the Bradford Hill criteria be investigated to determine whether there is causality. To date, no such “perfectly clear-cut” association between glyphosate exposure and any cancer exists. However, investigative toxicology is an important discipline to evaluate chemicals before any human exposure occurs, and these data may inform subsequent considerations of whether associations are attributable to causality. One Bradford Hill criterion in establishing disease causality is plausibility, based on known disease etiologies. In the case of lymphoma, there are numerous etiologies for the numerous and different lymphoma diseases, and as such, each lymphoma type should be investigated for a plausible mechanism to determine whether causality may be attributed an appropriately qualified association. Another Bradford Hill criterion is identification of a biological gradient, or dose-response, which is a key consideration in the following data evaluation.

## Chronic toxicity studies

Several one-year chronic studies have been undertaken in dogs and one in rats, in addition to the many chronic/carcinogenicity studies with one-year interim sacrifice groups. Current Test Guidelines (OECD, EPA, EU and JMAFF) for long-term studies clearly state that the highest dose tested should either be at the maximum tolerated dose (MTD), conventionally interpreted as a dose causing non-lethal toxicity, often noted as reduced body weight gain of 10% or more (IUPAC 1997). For test substances with low toxicity, a top dose not exceeding 1000 mg/kg bw/day may apply, except when human exposure indicates the need for a higher dose level to be used (OECD 2012a). All human exposure estimates are well below 1 mg/kg bw/day (see Discussion section), so that 1000 mg/kg bw/day is a practical limit dose for glyphosate in carcinogenicity studies. In the original pre-guideline chronic/carcinogenicity study, rats were dosed well below the MTD (Monsanto 1981), but in many subsequent studies, they were dosed well in excess of today's standard practice of not exceeding the dose limit.

### Dog chronic studies

Five one-year oral toxicity studies have been conducted in Beagle dogs (Table 2). Studies in dogs are not designed to detect neoplastic effects; these studies are therefore not discussed in detail. Nonetheless, the histopathological investigations that are part of one-year dog studies according to OECD TG 452 did not identify (pre) neoplastic lesions related to the administration of glyphosate.

Treatment-related effects in dog studies with glyphosate were restricted to non-specific findings like small retardations in body weight gain and soft stools, which are common findings in this test species. The lowest relevant NOAEL (i.e. highest NOAEL below the lowest LOAEL) in dogs on a daily treatment regimen for one year was 500 mg/kg bw/day. These studies demonstrate that glyphosate is of very low toxicity following repeat exposures in dogs.

### Rat chronic studies

The chronic toxicity potential of glyphosate acid was assessed in a 12-month feeding study (conducted in 1995 and 1996) in 24 male and female Wistar rats per group, dosed at 0, 2000, 8000 and 20 000 ppm (Syngenta 1996). The mean achieved dose levels were 0, 141, 560 and 1409 mg/kg bw/day for males, and 0, 167, 671 and 1664 mg/kg bw/day for females. Spastically significant reductions in bodyweight were evident in animals receiving 20 000 ppm glyphosate acid, together with a marginal reduction in bodyweight in rats receiving 8000 ppm, but food consumption relative to controls was lower for these dose groups, suggesting reduced palatability of the diets containing these doses of glyphosate. There were no toxicologically significant or treatment-related effects on hematology, blood and urine clinical chemistry, or organ weights (Table 2).

The treatment-related pathological finding, that is increased incidence of mild focal basophilia, and a hypertrophy of the acinar cells of the parotid salivary gland in both sexes which had received 20 000 ppm glyphosate acid, is considered an adaptive response due to oral irritation from the ingestion of glyphosate, an organic acid, in the diet. This was verified by mode of action investigations and studies with dietary administration of citric acid, a non-toxic organic acid with irritation properties and pH dilution curve similar to those of glyphosate (Saltmiras et al. 2011), which elicited the same response in the acinar cells of the parotid salivary glands.

In conclusion, the 12-month NOAEL in rats for glyphosate acid, as determined from this study, is 8000 ppm (corresponding to 560 mg/kg bw/day in males and 671 mg/kg bw/day in females). This study does not cover neoplastic endpoints. These were addressed in a subsequent study by the same sponsor (Syngenta 2001). Consistent with the findings observed in dogs, this study demonstrates that glyphosate is of very low toxicological concern following long-term daily exposures.

Similarly, most of the following 2-year rat carcinogenicity studies included additional groups for 1-year interim sacrifice to evaluate chronic toxicity. These studies did not elucidate significant toxicological concerns for chronic dietary exposures to glyphosate in rats in multiple expert reviews by governmental agencies and several technical branches of the World Health Organization including the Joint Meeting on Pesticide Residues Toxicological Evaluations (WHO/FAO 2004a).

## Carcinogenicity studies

Chronic/carcinogenicity tests are designed to simulate lifetime exposures to an individual chemical and represent the most robust *in vivo* assay to evaluate the effects of chronic exposure including carcinogenicity. These models are biological systems with natural background variability due to tumor formation as a natural consequence of aging. Glyphosate was found to have no carcinogenic potential, which is reflected in the data showing only background noise of spontaneous tumors across the wide range of doses. Normal biological variability should display various tumor types across all dose groups without an apparent dose-response. The study summaries discuss “select neoplasms”, identified by the authors as having an elevated incidence above concurrent controls across one or more dose groups, most of which lacked statistical significance and/or dose-response within an individual study. These tumors are then evaluated in the context of the whole data set, to provide a robust weight of evidence overview for the doses spanning several orders of magnitude. While not all studies have select neoplasms identified in the individual study summary tables, select neoplasms for all studies are reported in Tables 20–23. Summary tables of the select neoplasms footnote the strain tested for each dose, to allow consideration of strain differences in spontaneous tumor susceptibility (Tables 20–23). In addition, complete tumor incidence summary tables have been extracted from the original eight rat (the published rat study, Study 9, is not included) and five mouse study reports or study files, and posted in their original format, as a comprehensive online data supplement to this manuscript.

**Table 20.** Summary of select neoplasms in male rats (Studies 1–8).

	Tumor Incidence/number of animals examined, by dose (mg/kg bw/day)													
Select neoplasm	Controls – 0 [% range for studies]			^a^3	^d^7.4	^a^10	^c^10	^a^31	^d^73.9	^h^86	^b^89	^c^100	^f^104	^g^121
Pancreas islet cell adenoma	20/397 [0–14]			5/49	0/30	2/50	1/24	2/50	0/32	1/51	8/57	2/17	1/75	2/64
Pituitary adenoma	153/398 [6–57]			19/49	4/30	20/48	12/24	18/47	3/31	11/51	32/58	8/19	41/75	17/63
Pituitary carcinoma	4/98 [2–6]			2/49	NF	3/48	1/24	1/47	NF	NF	NF	0/19	NF	NF
Testes interstitial cell (Leydig)	14/447 [0–8]			3/50	0/37	1/50	1/25	6/50	2/32	3/51	0/60	0/19	2/75	2/63
Thyroid C cell adenoma	35/391 [4–18]			1/49	0/26	0/49	1/21	2/49	1/29	^#^1/51	5/58	1/17	10/74	^#^1/63
Hepatocellular adenoma	30/351 [0–48]			NF	22/50	NF	1/50	NF	10/48	2/51	2/60	1/49	0/75	2/64
Hepatocellular carcinoma	22/384 [0–42]			0/50	28/50	1/50	1/50	2/50	18/48	0/51	2/60	1/49	1/75	NF
Benign keratoacanthoma (skin)	8/250 [2–5]			NF	NF	NF	NF	NF	NF	3/51	3/60	NF	3/75	0/64

	Tumor Incidence/number of animals examined, by dose (mg/kg bw/day)													
Select neoplasm	^e^150	^h^285	^c^300	^f^354	^g^361	^b^362	^d^740.6	^e^780	^b^940	^c^1000	^h^1077	^f^1127	^g^1214	^e^1290
Pancreas islet cell adenoma	NF	2/51	2/21	1/80	0/64	5/60	1/49	NF	7/59	1/49	1/51	1/78	1/64	NF
Pituitary adenoma	NF	10/51	7/21	33/80	18/64	34/58	5/49	NF	32/59	17/50	20/51	42/78	19/63	NF
Pituitary carcinoma	NF	NF	1/21	NF	NF	NF	NF	NF	NF	0/50	NF	NF	NF	NF
Testes interstitial cell (Leydig)	1/49	1/51	0/21	0/80	2/63	3/60	3/50	2/49	2/60	2/50	1/51	2/78	2/64	0/47
Thyroid C cell adenoma	NF	^#^0/51	2/21	5/79	^#^1/63	8/58	1/50	NF	7/60	8/49	^#^3/51	6/78	^#^0/64	NF
Hepatocellular adenoma	NF	0/51	2/50	2/80	0/64	3/60	21/50	NF	8/60	2/50	1/51	1/78	5/64	NF
Hepatocellular carcinoma	1/49	0/51	0/50	2/80	NF	1/60	24/50	0/49	2/60	0/50	0/51	1/78	NF	0/47
Benign keratoacanthoma (skin)	NF	0/51	NF	0/80	1/64	4/60	NF	NF	5/59	NF	6/51	7/78	1/63	NF

^a^Study 1 (Monsanto) (CD) SD rats, rated unreliable for carcinogenicity evaluation.

^b^Study 2 (Monsanto) (CD) SD rats, including interim sacrifice groups.

^c^Study 3 (Cheminova) SD rats.

^d^Study 4 (Feinchemic Schwebda) Wistar rats.

^e^Study 5 (Excel) SD rats, rated unreliable for carcinogenicity evaluation.

^f^Study 6 (Arysta Life Sciences) Crj:CD SD rats, including interim sacrifice groups.

^g^ Study 7 (Syngenta) Alpk:AP_f_SD Wistar rats, including interim sacrifice groups.

^h^ Study 8 (Nufarm) Wistar Han Crl:WI rats.

^#^Recorded as parafollicular adenoma.

*NF* not found/not reported

**Table 21.** Summary of select neoplasms in female rats (Studies 1–8).

	Tumor Incidence/number of animals examined, by dose (mg/kg bw/day)													
Select neoplasm	Controls – 0 [% range for studies]			^a^3	^d^7.4	^c^10	^a^11	^a^34	^d^73.9	^c^100	^h^105	^b^113	^f^115	^g^145
Pancreas islet cell adenoma	11/397 [0–9]			1/50	0/23	2/27	1/50	0/49	0/16	2/29	0/51	1/60	2/79	0/63
Pituitary adenoma	246/397 [14–78]			29/48	13/33	19/28	31/50	26/49	7/23	19/29	23/51	48/60	54/79	44/63
Pituitary carcinoma	16/155 [2–17]			7/48	NF	5/28	5/50	12/49	NF	5/28	NF	0/60	NF	NF
Thyroid C cell adenoma	25/302 [3% – 16%]			3/49	0/24	1/27	6/50	3/47	1/17	1/29	^#^ 1/51	2/60	7/78	^#^ 0/63
Hepatocellular adenoma	22/302 [0–36]			NF	18/48	1/50	NF	NF	19/49	3/50	0/51	2/60	1/79	0/64
Hepatocellular carcinoma	14/210 [0–20]			0/50	15/48	0/50	0/50	2/50	14/49	0/50	0/51	0/60	NF	NF
Mammary gland fibroadenoma	113/384 [6–58]			16/46	NF	12/28	20/48	16/44	NF	17/29	9/51	^$^24/54	30/79	4/63
Mammary gland adenocarcinoma	40/334 [2–22]			6/46	0/30	NF	5/48	8/44	0/33	NF	3/51	^∼^10/54	8/79	0/63

	Tumor Incidence/number of animals examined, by dose (mg/kg bw/day)													
Select neoplasm	^e^210	^c^300	^h^349	^f^393	^g^437	^b^457	^d^740.6	^c^1000	^e^1060	^b^1183	^f^1247	^h^1382	^g^1498	^e^1740
Pancreas islet cell adenoma	NF	2/29	0/51	1/78	1/64	4/60	1/49	1/49	NF	0/59	1/78	0/51	0/64	NF
Pituitary adenoma	NF	25/30	16/51	47/77	46/63	46/60	6/50	34/49	NF	34/59	52/78	32/51	49/64	NF
Pituitary carcinoma	NF	2/30	NF	NF	NF	0/60	NF	7/49	NF	1/59	NF	NF	NF	NF
Thyroid C cell adenoma	NF	2/29	^#^1/50	8/76	^#^0/64	6/60	1/47	7/49	NF	6/60	4/78	^#^ 0/51	^#^ 2/64	NF
Hepatocellular adenoma	NF	1/50	1/51	0/78	1/64	6/60	13/50	2/50	NF	1/60	0/78	1/51	0/64	NF
Hepatocellular carcinoma	NF	0/50	1/51	NF	NF	1/60	9/50	0/50	NF	2/60	NF	0/51	NF	NF
Mammary gland fibroadenoma	1/22	19/30	7/51	27/77	6/64	^$^27/59	NF	29/50	5/22	^$^28/57	30/78	5/51	5/64	5/50
Mammary gland adenocarcinoma	0/22	NF	1/51	11/77	0/64	^∼^14/59	0/48	NF	0/22	^∼^9/57	8/78	6/51	2/64	0/50

^a^Study 1 (Monsanto) (CD) SD rats, rated unreliable for carcinogenicity evaluation.

^b^Study 2 (Monsanto) (CD) SD rats, including interim sacrifice groups.

^c^Study 3 (Cheminova) SD rats.

^d^Study 4 (Feinchemic Schwebda) Wistar rats.

^e^Study 5 (Excel) SD rats, rated unreliable for carcinogenicity evaluation.

^f^Study 6 (Arysta Life Sciences) Crj:CD SD rats, including interim sacrifice groups.

^g^Study 7 (Syngenta) Alpk:AP_f_SD Wistar rats, including interim sacrifice groups.

^h^Study 8 (Nufarm) Wistar Han Crl:WI rats.

^$^Recorded as adenoma/adenofibroma/fibroma.

^∼^Recorded as carcinoma/adenocarcinoma.

*NF* not found/not reported.

**Table 22.** Summary of select neoplasms in male mice (Studies 10–14).

	Tumor Incidence/number of animals examined, by dose (mg/kg bw/day)							
Select neoplasm	Controls – 0 [% range for studies]	^d^14.5	^e^85	^b^100	^d^150	^a^157	^c^165	^e^267
Bronchiolar-alveolar adenoma	31/249 [10–18]	2/22	^§^7/51	15/50	0/22	9/50	^§^14/50	^§^9/51
Bronchiolar-alveolar adenocarcinoma	10/149 [2–10]	NF	^§^5/51	NF	NF	3/50	^§^1/50	^§^7/51
Bronchiolar-alveolar carcinoma	10/100 [0–20]	0/22	NF	7/50	0/22	NF	NF	NF
Hepatocellular adenoma	27/250 [0–28]	5/25	1/51	12/50	3/28	0/50	15/50	4/51
Hepatocellular carcinoma	15/250 [0–16]	0/25	11/51	5/50	0/28	0/50	1/50	7/51
Malignant lymphoma	16/205 [0–100]	15/50	1/51	2/4	16/50	^#^5/50	2/50	2/51
Myeloid leukemia	3/101 [0–6]	1/50	1/51	NF	1/50	NF	NF	0/51

	Tumor Incidence/number of animals examined, by dose (mg/kg bw/day)							
Select neoplasm	^b^300	^a^814	^c^838	^e^946	^b^1000	^d^1454	^c^4348	^a^4841
Bronchiolar-alveolar adenoma	11/50	9/50	^§^13/50	^§^4/51	13/50	1/50	^§^11/50	9/50
Bronchiolar-alveolar adenocarcinoma	NF	2/50	^§^6/50	^§^11/51	NF	NF	^§^4/50	1/50
Bronchiolar-alveolar carcinoma	8/50	NF	NF	NF	9/50	1/50	NF	NF
Hepatocellular adenoma	11/50	1/50	15/50	2/51	9/50	3/50	7/50	0/50
Hepatocellular carcinoma	6/50	0/50	3/50	4/51	7/50	2/50	1/50	2/50
Malignant lymphoma	1/1	^#^4/50	0/50	5/51	6/8	19/50	6/50	^#^2/50
Myeloid leukemia	NF	NF	NF	0/51	NF	1/50	NF	NF

^a^Study 10 (Monsanto) CD-1 mice.

^b^Study 11 (Cheminova) CD-1 mice.

^c^Study 12 (Arysta Life Science) CD-1 mice.

^d^Study 13 (Feinchemic Schwebda) Swiss albino mice.

^e^Study 14 (Nufarm) CD-1 mice.

^§^Recorded as lung rather than bronchiolar-alveolar.

^#^Recorded as sum of malignant lymphoblastic lymphosarcoma with leukemia, lymphoblastic lymphosarcoma without leukemia and composite lymphosarcoma.

^$^Recorded as lymphoblastic lymphosarcoma with leukemia.

*NF* not found/not reported.

**Table 23.** Summary of select neoplasms in female mice (Studies 10–14).

	Tumor incidence/number of animals examined, by dose (mg/kg bw/day)							
Select neoplasm	Controls – 0 [% range for studies]	^d^15.0	^e^85	^b^100	^d^151	^c^153	^a^190	^e^267
Bronchiolar-alveolar adenoma	28/250 [2–20]	0/16	^§^4/51	3/49	2/21	^§^5/50	9/50	^§^2/51
Bronchiolar-alveolar adenocarcinoma	2/99 [2]	NF	^§^2/51	NF	NF	^§^2/50	3/50	^§^2/51
Bronchiolar-alveolar carcinoma	9/151 [2–10]	0/16	NF	2/49	0/20	NF	NF	NF
Malignant lymphoma	54/215 [10–100]	20/50	8/51	12/15	19/50	4/50	^#^6/50	10/51
Myeloid leukemia	2/156 [0–4]	1/50	0/51	NF	2/50	0/50	NF	1/51
Pituitary adenoma	1/232 [0–2]	0/16	1/51	0/32	0/17	1/50	0/21	0/51

	Tumor incidence/number of animals examined, by dose (mg/kg bw/day)							
Select neoplasm	^b^300	^c^787	^e^946	^a^955	^b^1000	^d^1467	^c^4116	^a^5874
Bronchiolar-alveolar adenoma	3/50	^§^12/50	^§^2/51	10/49	6/50	3/50	^§^5/50	1/50
Bronchiolar-alveolar adenocarcinoma	NF	^§^3/50	^§^3/51	4/49	NF	NF	^§^1/50	4/50
Bronchiolar-alveolar carcinoma	1/50	NF	NF	NF	5/50	0/50	NF	NF
Malignant lymphoma	9/12	8/50	11/51	^#^6/50	13/14	25/50	7/50	^#^10/50
Myeloid leukemia	NF	0/50	0/51	NF	NF	1/50	1/50	NF
Pituitary adenoma	0/23	0/50	2/51	0/44	^∼^3/50	1/48	0/50	0/37

^a^Study 10 (Monsanto) CD-1 mice.

^b^Study 11 (Cheminova) CD-1 mice.

^c^Study 12 (Arysta Life Science) CD-1 mice.

^d^Study 13 (Feinchemic Schwebda) Swiss albino mice.

^e^Study 14 (Nufarm) CD-1 mice.

^§^Recorded as lung rather than bronchiolar-alveolar.

^#^Recorded as sum of lymphoblastic lymphosarcoma with leukemia, lymphoblastic lymphosarcoma without leukemia and composite lymphosarcoma.

^∼^2 animals in anterior lobe, 1 animal in intermediate lobe.

*NF* not found/not reported.

## Rat carcinogenicity

A total of nine chronic/carcinogenicity studies in the rat, including one peer-reviewed published study, were available for review. This duplication of large-scale studies in the same animal model using the same test substance is not consistent with today's broader appreciation for animal welfare and the reduction of unnecessary animal testing. However, these studies offer the opportunity for a critical discussion of findings in individual studies in the context of the larger body of data. Wistar and Sprague Dawley were the strains used for the bioassays in rats. Seven studies were conducted under conditions of GLP, and two studies were not under GLP (Study 1, conducted before the introduction of GLP; Study 9, non-GLP). Most studies in rats were designed as combined chronic toxicity/ carcinogenicity studies, with interim sacrifices after 12 months of treatment for the assessment of non-neoplastic chronic toxicity. Statistical methods are noted in the manuscript tables where statistical significance was attained. Statistical differences in neoplasm incidence summary tables are reported in the online data supplements. Chronic endpoints and NOAEL values are captured in each study summary table; however, the following study reviews focus on carcinogenicity.

### Study 1 (Monsanto 1981)

An early study into the long-term effects of orally administered glyphosate in the rat was conducted between 1978 and 1980 (Monsanto 1981), prior to the adoption of international test guidelines and GLP standards (Tables 3–6). Nonetheless, the test protocol was broadly compliant with OECD TG 453 (1981). However, an MTD was not reached and the high dose was well below an acceptable dose limit of 1000 mg/kg bw/day. Therefore, this study is rated Klimisch 3 for reliability, and is considered inadequate for carcinogenicity evaluation from a regulatory perspective.

Groups of 50 male and 50 female Sprague Dawley rats were administered glyphosate acid in the diet, at concentrations of 0, 30, 100 and 300 ppm, for up to least 26 months. The mean doses achieved were 0 (control), 3, 10, and 31 mg/kg bw/day for the males, and 0 (control), 3, 11, and 34 mg/kg bw/day for the females. Study results are summarized in Table 3.

In general, the incidences of all neoplasms observed in the treated and control animals were similar, or occurred at low incidence, such that a treatment-related association could not be made. The most common tumors found were common spontaneous neoplasms, as reported in the literature relating to rat (Johnson and Gad 2008), in the pituitary glands of both control and treated animals (Table 4). In the females, mammary gland tumors were the next most common neoplasm across control and dose groups (see data Supplementary Study 1 to be found online at http://informahealthcare.com/doi/abs/10.3109/10408444.2014.1003423).

The incidence of interstitial cell tumors of the testes in male rats in both the scheduled terminal sacrifice animals, as well as for all animals, suggested a possible treatment-related finding, and was presented along with contemporary historical control data for comparison (Tables 5 and 6). It was noted that at 12 months, the incidence of interstitial tumors was near zero; however, in animals aged 24–29 months at necropsy, the incidence increased to approximately 10%. The historical control data for chronic toxicity and carcinogenicity from 5 studies terminated at 24–29 months showed background levels of interstitial cell tumors comparable to those found at the highest dose in the study. Furthermore, the reported incidences in all dose groups reflect the normal range of interstitial cell tumors in rat testes, reported in the Registry of Industrial Toxicology Animal Data (Nolte et al. 2011). The incidence of interstitial cell hyperplasia did not provide evidence of a pre-neoplastic lesion. The investigators noted that at terminal sacrifice, the incidence of interstitial cell tumor was 15.4% (4/26), while the range in control animals from 5 contemporary studies (historical controls) was 6.2% (4/65) to 27.3% (3/11), with an overall mean value of 9.6% (16/166). When all animals on test are included, the incidence for the high-dose males was 12% (6/50), compared to a contemporary historical control range of 3.4% (4/116) to 6.7% (5/75), with a mean of 4.5% (24/535). The concurrent control incidence of interstitial cell tumors (0%) was not representative of the normal background incidence noted in contemporary historical control data. Therefore, the data suggest that the incidence in treated rats is within the normal biological variation observed for interstitial cell tumors at this site in this strain of rat. When evaluated in the context of the full data set for male rats (Table 20), a dose-response is clearly absent for the 25 doses evaluated in rats, ranging from 3 to 1290 mg/kg bw/day, which demonstrates that this tumor is clearly not a consequence of glyphosate exposure.

In conclusion, glyphosate was not considered carcinogenic in Sprague Dawley rats following continuous dietary exposure of upto 300 ppm, corresponding to 31 and 34 mg/kg bw/day in males and females, respectively, which is consistent with evaluations by the US EPA (US EPA 1993), the original Annex I listing in Europe (EC 2002), and WHO/FAO (WHO/FAO 2004a).

Based on the low doses tested in Study 1, Monsanto was obliged to conduct a second chronic/carcinogenicity study in rats (Study 2, discussed below) in accordance with OECD TG 453 (1981), which had been developed and instituted after this initial study was conducted.

### Study 2 (Monsanto 1990)

In response to evolving regulatory requirements, this study was conducted in accordance with the contemporary version of OECD TG 453 (Monsanto 1990). The chronic toxicity and carcinogenic potential of glyphosate were assessed in a 24-month feeding study in 50 male and 50 female Sprague Dawley rats, dosed with 0, 2000, 8000 and 20 000 ppm (equivalent to mean achieved dose levels of 0, 89, 362 and 940 mg/kg bw/day for males and 0, 113, 457 and 1183 mg/kg bw/day for females (Table 7). In addition, 10 rats per sex per dose were included for interim sacrifice after 12 months. Observations covered clinical signs, ophthalmic examinations, body weight, food consumption, hematology, clinical chemistry and urinalysis, as well as organ weights, necropsy, and histopathological examination. This study was rated Klimisch 1 for reliability.

Treatment-related findings in this study were significantly reduced body weight in high-dose females, as well as increased liver weight in high-dose males and females, and a slight increase in incidence of cataract lens changes in high-dose males, which was not statistically significant for eye lesions confirmed by histopathology (Table 7). The body weight changes confirm that the MTD was achieved in the highest dose group. Benign thyroid C-cell adenomas were statistically higher than controls in the mid-dose terminally sacrificed males, but when pooled with unscheduled deaths, no statistically significant increase was noted. Benign pancreas islet cell adenomas were not statistically higher for the unscheduled or scheduled deaths, but when combined, were statistically higher than controls in the low and high dose males. In both cases, the benign tumors did not exhibit a dose-response, and did not progress to carcinomas, and thus the US EPA concluded that these tumors were not related to the administration of glyphosate (US EPA 1993). These neoplasms, in addition to skin keratoacanthoma in males, a common rat tumor, were selected for further weight of evidence evaluation (Tables 20 and 21). No evidence of a glyphosate-induced carcinogenic effect was noted in either sex (see data Supplementary Study 2 to be found online at http://informahealthcare.com/doi/abs/10.3109/10408444.2014.1003423).

In conclusion, glyphosate was not carcinogenic in Sprague Dawley rats following continuous dietary exposure of up to 20 000 ppm for 24 months, corresponding to 940 and 1183 mg/kg bw/day in males and females, respectively, which is consistent with evaluations by the US EPA (US EPA 1993), European Authorities (EC 2002), and WHO/FAO (WHO/FAO 2004a).

### Study 3 (Cheminova 1993a)

The chronic toxicity and carcinogenic potential of glyphosate technical acid were assessed in a 104-week feeding study in male and female Sprague Dawley rats (Cheminova 1993a). The study was conducted between 1990 and 1992. Groups of 50 rats per sex received daily dietary doses of 0, 10, 100, 300, or 1000 mg/kg bw/day of glyphosate technical acid for 24 months (Table 8). Five additional groups of 35 rats per sex, receiving daily dietary doses of, 0, 10, 100, 300 or 1000 mg/kg bw/day, were included for interim sacrifice at the 12th month for evaluation of chronic toxicity. The dietary glyphosate levels were adjusted weekly to ensure that animals were receiving the intended dose levels at all times. This study was rated Klimisch 1 for reliability.

At 1000 mg/kg bw/day, female mean liver weights were decreased, while males and females had statistically significant reductions in body weight throughout the study, confirming that the MTD was achieved (Table 8). Neoplasms were noted in control and treated groups, but dose-responses were not evident, and no statistically significant increases versus controls were noted for any tumor type (*p* < 0.05). No treatment-related neoplastic lesions were observed at termination, and no select neoplasms were identified in this study for further consideration (see data Supplementary Study 3 to be found online at http://informahealthcare.com/doi/abs/10.3109/10408444.2014.1003423). Glyphosate was not considered carcinogenic in male and female Sprague Dawley rats following 104 weeks of continuous dietary exposure of up to 1000 mg/kg bw/day, the limit dose, which is consistent with evaluations by the European Authorities (EC 2002, Germany Rapporteur Member State 2015b) and WHO/FAO (WHO/FAO 2004a).

### Study 4 (Feinchemie Schwebda 1996)

A 2-year bioassay in the Wistar rat used dietary glyphosate levels of 0, 100, 1000, and 10 000 ppm (Feinchemie Schwebda 1996). Groups of 50 rats per sex were fed for 24 months. The mean achieved dose levels were 0, 7.4, 73.9, and 740.6 mg/kg bw/day (Table 9). This study was rated Klimisch 1 for reliability.

In addition, one vehicle control with ten rats per sex and one high dose (10 000 ppm) group with 20 rats per sex were included for interim sacrifice after one year of treatment, to study non-neoplastic histopathological changes. The mean achieved dose level in the treated group was 764.8 mg/kg bw/day. Observations covered clinical signs, body weight, food consumption, hematology, clinical chemistry, and urinalysis, as well as organ weights, necropsy, and histopathological examination.

There were no treatment-related deaths or clinical signs in any of the dose-groups. Moreover, there were no treatment- related effects on body weight gain or food consumption noted. This suggests that the MTD may not have been reached by the applied dosing regimen.

There was some background variation in the incidences of benign tumors (e.g. reduced tumor incidence in low and middose males, increased tumor incidence in middose females), which was considered incidental in absence of a dose-response relationship (see data Supplementary Study 4 to be found online at http://informahealthcare.com/doi/abs/10.3109/10408444.2014.1003423).

The different liver tumors observed in the dead and moribund sacrificed and terminally sacrificed rats included hepatocellular adenoma, intrahepatic bile duct adenomas, cholangiocarcinoma, hepatocellular carcinoma, histiocytic sarcoma, fibrosarcoma, and lymphosarcoma. Among these, hepatocellular adenomas and carcinomas occurred more frequently, as often observed in aging rats (Thoolen et al. 2010). These tumors appeared to be incidental and not compound-related, as their frequency of occurrence was not dependent on dose. Hepatocellular adenomas and carcinomas were considered select neoplasms (Table 9), based on increased incidence above controls for total animals, albeit non-dose responsive, for adenoma in mid-dose females, carcinoma in low- and high-dose males, and carcinoma in low- and mid-dose females. These liver neoplasms are considered in the weight of evidence evaluation (Tables 20 and 21).

The study report concluded that glyphosate technical acid was not carcinogenic in Wistar rats following continuous dietary exposure of up to 595 and 886 mg/kg bw/day in males and females, respectively, for 24 months, which is consistent with evaluations by the European Authorities (EC 2002, Germany Rapporteur Member State 2015b).

### Study 5 (Excel 1997)

A 2-year feeding study in the Sprague Dawley rats (Excel 1997) featured dietary concentrations of 0, 3000, 15 000, and 25 000 ppm glyphosate technical acid. Groups of 50 rats per sex were fed for 24 months, and mean dose levels of 0, 150, 780 and 1290 mg/kg bw/day (males) and 0, 210, 1060 and 1740 mg/kg bw/day (females) were achieved (Table 10).

In addition, 20 rats/sex/group were included for interim sacrifice at week-52, to study non-neoplastic histopathological changes with a different high-dose level of 30 000 ppm. The dietary doses correspond to 180, 920 and 1920 mg/kg bw/day (males) and 240, 1130 and 2540 mg/kg bw/day (females), for 3000, 15 000 and 30 000 ppm, respectively. Thus, a limit dose above 1000 mg/kg bw/day was achieved.

The study report notes that glyphosate technical acid was not carcinogenic in Sprague Dawley rats following continuous dietary exposure to up to 1290 mg/kg bw/day, and 1740 mg/kg bw/day for males and females, respectively, for 24 months. However, this study was rated Klimisch 3 for reliability (Germany Rapporteur Member State 2015b), and therefore, is considered unreliable for carcinogenicity evaluation based on lower than expected background tumor incidences (see data Supplementary Study 5 to be found online at http://informahealthcare.com/doi/abs/10.3109/10408444.2014.1003423). In addition, the test substance was not adequately characterized, and several deviations from the OECD Test Guideline 453 were noted.

### Study 6 (Arysta Life Sciences 1997b)

A combined chronic toxicity/carcinogenicity study in Sprague Dawley rats (Arysta Life Sciences 1997b) was conducted between December 1994 and December 1996. The rats were fed 0, 3000, 10 000, and 30 000 ppm glyphosate for two years (equivalent to 0, 104, 354 and 1127 mg/kg bw/day for males and 0, 115, 393 and 1247 mg/kg bw/day for females (Table 11). Thus, a limit dose was achieved, and the MTD was noted at the high dose in males and females with decreased body weight, increased cecum weight, distention of the cecum, loose stool and skin lesions. In addition, 30 rats/sex/group were included for interim sacrifice at 26, 52 and 78 weeks, to study non-neoplastic histopathological changes. Observations covered clinical signs, body weight, food consumption, hematology, clinical chemistry, and urinalysis, as well as organ weights, necropsy, and histopathological examination. This study was rated Klimisch 1 for reliability.

Non-statistically significant increases versus controls (*p* < 0.05) were noted for pituitary adenomas, skin keratoacanthoma in high-dose males, and mammary gland fibroadenoma in low and mid-dose females (Table 11). These neoplasms were considered for the weight of evidence evaluation (Tables 20 and 21), and the full tumor summary data are available online (see data Supplementary Study 6 to be found online at http://informahealthcare.com/doi/abs/10.3109/10408444.2014.1003423). As mentioned under Study 1, pituitary and mammary tumors are common spontaneous neoplasms in aging rats (Johnson and Gad 2008), and skin keratoacanthoma is noted as one of the most common spontaneous benign neoplasms in male Sprague Dawley rats (Chandra et al. 1992). The study report concluded that glyphosate was not carcinogenic in Sprague Dawley rats following continuous dietary exposure to up to 30 000 ppm for 24 months, corresponding to 1127 mg/kg bw/day and 1247 mg/kg bw/day for males and females, respectively, which is consistent with the recent evaluation in Europe under the Annex I Renewal of glyphosate (Germany Rapporteur Member State 2015b).

### Study 7 (Syngenta 2001)

The same rat model that was used in the previously discussed 12-month chronic rat study (Syngenta 1996b) was also employed in a 2-year feeding study (Syngenta 2001). A group of 52 male and 52 female Wistar rats received 0, 2000, 6000 or 20 000 ppm via feed (Table 12). The mean achieved dose levels were 0, 121, 361 and 1214 mg/kg bw/day for males, and 0, 145, 437 and 1498 mg/kg bw/day for females. Thus, a limit dose was achieved. In addition, three satellite groups with 12 rats per sex each were included for interim sacrifice after 12 months of treatment, to investigate potential non-neoplastic histopathological changes. Observations covered clinical signs, body weight, food consumption, hematology, clinical chemistry, and urinalysis, as well as organ weights, necropsy, and histopathological examination. This study was rated Klimisch 1 for reliability.

Treatment-related findings in this study were found in the liver and kidney, and were confined to animals (predominantly males) fed 20 000 ppm glyphosate acid. There were a number of changes in males and females fed 20 000 ppm glyphosate acid, notably renal papillary necrosis, prostatitis, periodontal inflammation, urinary acidosis, and hematuria, which may be attributed to the acidity of the test substance. Slight increases in proliferative cholangitis and hepatitis were noted in males at 20 000 ppm. Despite the findings at 20 000 ppm, survival was better in males fed 20 000 ppm than in the controls and lower dose groups. This improved survival was associated with a decreased severity of renal glomerular nephropathy and a 5% reduction in body weight (see data Supplementary Study 7 to be found online at http://informahealthcare.com/doi/abs/10.3109/10408444.2014.1003423, for neoplastic and non-neoplastic findings).

A small increase in the incidence of hepatocellular adenoma was observed in males fed 20 000 ppm glyphosate acid. While not statistically significant using the Fisher's exact test, the difference was statistically significant for total male rats using the Peto Test for trend. However, there was no evidence of pre-neoplastic foci, no evidence of progression to adenocarcinomas, and no dose-response. In addition, the incidence was within the laboratory's historical control range for tumors of this type in the liver (Table 12). Therefore, the increased incidence was considered not to be related to treatment, yet these were considered select neoplasms (Table 12) and evaluated in context of the complete data set (Tables 20 and 21).

The study report concluded that glyphosate acid was not carcinogenic in the Wistar rats following continuous dietary exposure to up to 20 000 ppm for 24 months, at 1214 and 1498 mg/kg bw/day in males and females, respectively, which is consistent with the WHO/FAO review (WHO/FAO 2004a) and the recent evaluation in Europe under the Annex I Renewal of glyphosate (Germany Rapporteur Member State 2015b).

### Study 8 (Nufarm 2009b)

The most recent study in this series of regulatory studies investigating the potential carcinogenicity of glyphosate in rats was conducted from September 2005 through March 2008 (Nufarm 2009b). The study was conducted by feeding dietary concentrations of 0, 1500, 5000 and 15 000 ppm glyphosate to groups of 51 Wistar rats per sex. To ensure that a received limit dose of 1000 mg/kg bw/day overall was achieved, the highest dose level was progressively increased to 24 000 ppm. Mean dose levels of 86/105, 285/349, and 1077/1382 mg glyphosate/kg bw/day (males/females) were achieved (Table 13). This study was rated Klimisch 1 for reliability.

Non-neoplastic findings included transient liver enzyme activity for mid-dose males and high-dose males and females, and equivocal nephrocalcinosis depositions at the high-dose. Histopathology noted a statistically significant increase in adipose infiltration of the bone marrow in high-dose males compared to controls, suggestive of myeloid hypoplasia, which may be considered a stress response (Everds et al. 2013).

Skin keratoacanthoma in males and mammary gland adenocarcinoma in females (Table 13) were considered for evaluation in the context of the weight of evidence for rat tumor incidence (Tables 20 and 21), wherein dose-responses were not evident. Tumor incidence summary data have been tabulated (see data Supplementary Study 8 to be found online at http://informahealthcare.com/doi/abs/10.3109/10408444.2014.1003423). Microscopic evaluation of tissues did not reveal any indications of neoplastic lesions caused by glyphosate treatment. The study report concluded that glyphosate acid was not carcinogenic in Wistar rats following continuous dietary exposure to up to 24 000 ppm for 24 months, at 1077 and 1382 mg/kg bw/day in males and females, respectively, which is consistent with the recent evaluation in Europe under the Annex I Renewal of glyphosate (Germany Rapporteur Member State 2015b).

### Study 9 Publication (Chruscielska et al. 2000a)

A two-year combined chronic toxicity and carcinogenicity study in Wistar rats was published by academic researchers from Warsaw, Poland. The study was conducted as a drinking-water study in Wistar-RIZ rats according to OECD TG 453. The test material was a 13.85% aqueous formulation of glyphosate as its ammonium salt (equivalent to 12.6% glyphosate acid). However, the ammonium salt of glyphosate tested is not commercially available, and the concentration of active ingredient suggests that a glyphosate-formulated product was tested; this is supported by a concurrent genotoxicity publication by the same lead author (Chruscielska et al. 2000b), previously reviewed by Kier and Kirkland (Kier and Kirkland 2013), in which a glyphosate formulation, Perzocyd, was tested. Deficiencies noted with respect to OECD TG 453 include insufficient dosing to elicit toxic effects, inadequate test material characterization, no reporting of water/feed consumption, body weights and diet composition, and no individual animal data. Although the manuscript reporting deficiencies may have been included in the study, they were not reported in the manuscript, and could warrant a Klimisch reliability score of 4 (not assignable), but the low doses employed in this study justify a Klimisch reliability score of 3.

The test material was administered in water at glyphosate salt concentrations of 0, 300, 900, and 2700 mg/L. Each dose group consisted of 85 animals per sex. Ten animals per sex and dose were sacrificed after 6, 12, and 18 months of exposure, for evaluation of general toxicity. The remaining 55 animals per sex and dose were scheduled for sacrifice after 2 years of exposure.

Water consumption was claimed to have been measured, but these data have not been reported. To estimate the glyphosate doses received via drinking water, the assumed default water consumptions were 50 and 57 mL/kg bw/day by male and female rats, respectively (Gold et al. 1984). Using these standard figures and the glyphosate content of the tested formulation (12.6%), daily doses are estimated at 0, 1.9, 5.7, and 17 mg of glyphosate/kg bw/day for males and 0, 2.2, 6.5, and 19 mg of glyphosate/kg bw/day for females. As this study appears to have tested a formulated product, data were not included in the weight of evidence review (Tables 20 and 21), but given the very low glyphosate doses and reported low tumor incidence, these were of no consequence to the overall data review.

Exposure to glyphosate ammonium salt had no effect on body weight, appearance and behavior, and hematological parameters, which is consistent with glyphosate chronic toxicity data regulatory reviews. Even though there seems to be a trend towards higher 2-year mortality in treated females (Table 14), this difference had no statistical significance according to the authors. There were sporadic alterations of clinical-chemical and urinalysis parameters, but not in a consistent fashion over time and without dose-dependence. These alterations were not interpreted as treatment-related. There was no effect of glyphosate on the incidence of neoplastic lesions (Table 14). Thus, the NOAEL for chronic toxicity and carcinogenicity in this study was greater than or equal to 17 and 19 mg glyphosate/kg bw/day, in males and females, respectively.

Due to the lack of systemic effects in the highest dose group, the MTD was not reached by this study. Judging from other rat studies reviewed here, the MTD is likely to be greater than 1000 mg/kg bw/day. Thus, the top glyphosate dose of an estimated 19 mg/kg bw/day in this study is too low to satisfy regulatory validity criteria for a carcinogenicity study.

### Mouse carcinogenicity

There are a total of five carcinogenicity studies with glyphosate in mice, that have been submitted to support glyphosate Annex I renewal in the European Union. All but the oldest study (Study 10) were considered reliable without restriction, and were performed under conditions of GLP following OECD TGs. Most studies were conducted in the CD-1 strain. Each study was sponsored by a different manufacturer. In each case, technical grade glyphosate was administered via diet for at least 18 months. Select neoplasms, mostly lymphoreticular, liver and lung, are summarized for all mouse chronic studies in Tables 22 and 23. These neoplasms are widely recognized as occurring spontaneously in aging mice (Gad et al. 2008, Son and Gopinath 2004). Lymphomas have been recognized for many years as one of the most common, if not the most common category of spontaneous neoplastic lesions in aging mice (Brayton et al. 2012, Gad et al. 2008, Son and Gopinath 2004). The subclassification of malignant lymphomas is not a typical diagnostic feature in rodent studies, likely due to either expense and/or feasibility. It is, however, important to recognize that lymphomas are not a single type of neoplasm, rather they are a grouping of different neoplasms arising from different pathogeneses, and should be considered as different diseases (Bradley et al. 2012). As is the case for NHL in humans, these different immune system neoplasms are clustered together based on manifestation in lymphocytes, despite their very different etiologies; for example, the most common subset of NHL lymphomas clustered together as “diffuse large B cell lymphomas”, have for many years been considered multiple clinical-pathologic entities (Armitage 1997), and therefore may be considered attributable to different modes of action. Chronic endpoints and NOAEL values are captured in each study summary table; however, the following study reviews focus on carcinogenicity.

### Study 10 (Monsanto 1983)

The first chronic-carcinogenicity mouse study with glyphosate was conducted between March 1980 and March 1982 (Monsanto 1983), prior to the institution of GLP (Table 15). The study design was essentially in compliance with OECD TG 451 for carcinogenicity studies, adopted in 1981, when the study was already ongoing. Groups of 50 male and female CD-1 mice received glyphosate at dietary levels of 1000, 5000, and 30 000 ppm, over a period of nearly two years. The mean achieved doses were 157/190, 814/955, and 4841/5874 mg/kg bw/day in males and females, respectively, exceeding the limit dose. Based on this study predating both GLP and OECD TG 451, a reliability score of Klimisch 2 has been assigned.

In addition to post-mortem pathological examinations after terminal sacrifice, hematological investigations were performed on 10 mice per sex and dose at months 12 and 18, and on 12 male animals/group, as well as all surviving females at scheduled termination.

Two non-neoplastic histological changes affecting the liver and urinary bladder were assumed to be treatment-related. There was a higher incidence of centrilobular hepatocyte hypertrophy in high-dose males, and a more frequent occurrence of slight-to-mild bladder epithelial hyperplasia in the mid and high dose; however, a clear dose-response was lacking. Tumor incidences, which did not significantly increase with dose, were mostly bronchiolar-alveolar, hepatocellular, or lymphoreticular, all of which are commonly noted spontaneously occurring tumors in aging mice (Table 15). Lymphoreticular tumors combined for males and females totaled 7, 12, 10 and 12 for control, low, mid- and high-dose groups respectively, and were not considered as being related to test substance.

A more frequent occurrence of slight-to-mild bladder epithelial hyperplasia was observed in the mid and high-dose groups; however, clear dose-response was lacking (Table 15) and no urinary bladder neoplasms were noted at these doses (see data Supplementary Study 10 to be found online at http://informahealthcare.com/doi/abs/10.3109/10408444.2014.1003423). Benign renal tubule adenomas were noted in mid- and high-dose males at incidences of 1/50 and 3/50 respectively. These neoplasms were not observed in females, lacked statistical significance, and were considered spontaneous and unrelated to glyphosate administration by the study pathologists; this neoplasm, while not seen in the concurrent control group, had previously been noted in control male CD-1 mice of comparable age by the author of the study. As an additional measure of diligence, a Pathology Working Group was convened, and it concluded that the absence of any pre-neoplastic kidney lesion in all male animals provided sufficient evidence that this finding was spurious and not related to glyphosate administration. This is reflected in the US EPA review of glyphosate (US EPA 1993). This neoplasm was not observed in the other four mouse carcinogenicity studies discussed.

The author of the study also reported a trend towards a non-statistically significant increased occurrence of lymphoreticular neoplasia in treated female mice (Table 15). However, these consisted of three different categories of lymphoreticular neoplasms. Regulatory reviews confirmed that there is no apparent dose-dependence for these endpoints (EC 2002, US EPA 1993, WHO/FAO 2004a). Summary tables of incidence of neoplastic findings are available (see data Supplementary Study 10 to be found online at http://informahealthcare.com/doi/abs/10.3109/10408444.2014.1003423).

Glyphosate was reported as not carcinogenic in CD-1 mice up to doses well in excess of the limit dose for carcinogenicity testing, which is consistent with evaluations by the US EPA (US EPA 1993), European Commission (EC 2002), recent EU Annex I Renewal evaluation by the Rapporteur (Germany Rapporteur Member State 2015b), and WHO/FAO (WHO/FAO 2004a).

### Study 11 (Cheminova 1993b)

Another carcinogenicity bioassay in mice was conducted between December 1989 and December 1991 (Table 16) (Cheminova 1993b). In this assay, 50 male and 50 female CD-1 mice per dose group received glyphosate via their diet over a period of approximately two years. This treatment period is 6 months longer than the 18 months stipulated for mice by OECD TG 451 (1981 version). The dietary levels were adjusted regularly to achieve constant dose levels of 0, 100, 300 and 1000 mg/kg bw/day, achieving the limit dose. This study was rated Klimisch 1 for reliability.

Slight non-statistically significant increases in bronchiolar-alveolar adenomas were noted for all male dose groups above controls in a non-dose-responsive manner. Bronchiolar-alveolar neoplasms are evaluated in the context of the full data set (Tables 22 and 23), demonstrating a lack of dose-response across doses ranging from approximately 15 mg/kg bw/day to 5000 mg/kg bw/day. Although the number of pituitary adenomas were low and considered incidental, they were conservatively included in the select neoplasms, based on being slightly higher in high dose females than concurrent controls (Table 16). The data summary of all histological findings, including tumor incidence, is available (see data Supplementary Study 11 to be found online at http://informahealthcare.com/doi/abs/10.3109/10408444.2014.1003423).

There were no statistically significant increases in the occurrence of any tumor type in this study. The observed variations did not show a dose relationship, and were within the range of historical control data. Glyphosate was determined to be not carcinogenic to CD-1 mice at up to 1000 mg/kg bw/day, which is consistent with evaluations by the European Commission (EC 2002) and WHO/FAO (WHO/FAO 2004a).

### Study 12 (Arysta Life Sciences 1997a)

An 18-month feeding study in ICR-CD-1 mice, conducted between February 1995 and September 1996, investigated higher doses by admixing 1600, 8000, or 40 000 ppm glyphosate into the diet fed to groups of 50 male and 50 female mice per dose (Arysta Life Sciences 1997a). The calculated test substance intake was 165/153, 838/787, and 4348/4116 mg/kg bw/day (males/females, Table 17), exceeding the limit dose. This study was rated Klimisch 1 for reliability.

Histopathological examinations did not show statistically significant increases for any type of neoplastic lesion in all treatment groups of both sexes (see data Supplementary Study 12 to be found online at http://informahealthcare.com/doi/abs/10.3109/10408444.2014.1003423). Select neo- plasms evaluated across the data set with some non- statistically significant increases above concurrent controls included lymphoma and lung tumors, all of which lacked a clear dose-response. Glyphosate was considered not carcinogenic in CD-1 mice up to doses well in excess of the limit dose for carcinogenicity testing, which is consistent with the recent evaluation in Europe under the Annex I Renewal of glyphosate (Germany Rapporteur Member State 2015b).

### Study 13 (Feinchemie Schwebda 2001)

An 18-month feeding study in Swiss albino mice (Feinchemie Schwebda 2001), conducted between December 1997 and June 1999, featured treatment groups, each with 50 animals per sex, receiving 100, 1000, and 10 000 ppm technical grade glyphosate in the diet. Control mice received a plain diet. The calculated test substance intake was 14.5/15.0, 150/151, 1454/1467 mg/kg bw/day (males/females, Table 18), exceeding the limit dose, as reflected in elevated mortality in the high dose groups. This study was rated Klimisch 2 for reliability, based on speculation of a viral infection within the colony, discussed below.

Based on the slightly higher mortality and lower survival rates in the high dose groups, the NOAEL was considered 1000 ppm (151 mg/kg bw/day). There were no treatment-related effects on clinical signs, behavior, eyes, body weight, body weight gain, food consumption, and differential white blood cell counts in both sexes. Gross pathology, organ weight data, and histopathological examination demonstrated no treatment-related effects. An increase in the number of malignant lymphomas, the most common spontaneously occurring tumor category in the mouse, was statistically significant in the high-dose groups compared to controls (Table 18). The Germany Rapporteur Member State concluded that the malignant lymphoma increase in high-dose males was inconclusive but unrelated to treatment in the context of similar higher dosed studies (Germany Rapporteur Member State 2015b), and considered this endpoint irrelevant to carcinogenic risk in humans (Germany Rapporteur Member State 2015a). Whether or not a viral component (Taddesse-Heath et al. 2000) may have contributed to this endpoint, the finding was considered incidental background variation based on historical control data, and in agreement with the study director. As in Study 11, bronchiolar-alveolar adenoma was also considered a select neoplasm for evaluation in the broader data set (Tables 22 and 23), and as previously discussed, demonstrates a lack of dose-response across doses ranging from approximately 15 mg/kg bw/day to 5000 mg/kg bw/day. Summary tables of all histopathological neoplastic findings are available (see data Supplementary Study 13 to be found online at http://informahealthcare.com/doi/abs/10.3109/10408444.2014.1003423).

Technical grade glyphosate was reported as not carcinogenic in Swiss albino mice, following continuous dietary exposure of up to 1460 mg/kg bw/day (average for both sexes) for 18 months. The NOAEL for general chronic toxicity was 151 mg/kg bw/day for both sexes combined.

### Study 14 (Nufarm 2009a)

The most recent mouse carcinogenicity assay was conducted between October 2005 and November 2007 (Nufarm 2009a). Groups of 51 CD-1 mice per sex received daily dietary doses of 0, 500, 1500, and 5000 ppm technical grade glyphosate (equivalent to an average intake of 85, 267 and 946 mg/kg bw/day, Table 19). The MTD was apparently not reached in the high-dose group, which is more indicative of low general toxicity of the test substance rather than a flaw in the study design. The NOAEL for chronic toxicity was 810 mg/kg bw/day for male mice and 1081 mg/kg bw/day for female mice, the highest dosage tested. Despite not quite achieving a limit dose in males, this study was arguably rated Klimisch 1 for reliability.

Several increases in common spontaneous mouse neoplasms in male mice were noted. Non-dose-response increases were noted for hepatocellular adenoma and carcinoma in males, and dose-responses were noted for bronchiolar-alveolar adenocarcinoma and malignant lymphoma in males, but not females. Pituitary adenoma incidences were low, and considered incidental in low and high-dose females, although they were slightly higher than controls (Table 19). These neoplasms were all evaluated in context of the broader data set (Tables 22 and 23). The summary of neoplastic findings is available (see data Supplementary Study 14 to be found online at http://informahealthcare.com/doi/abs/10.3109/10408444.2014.1003423).

Glyphosate was considered not carcinogenic in the CD-1 mice, following continuous average dietary exposure for males and females, to quantities up to 945.6 mg/kg bw/day for 18 months, which is consistent with the recent evaluation in Europe under the Annex I Renewal of glyphosate (Germany Rapporteur Member State 2015b).

## Discussion

An extraordinarily large volume of animal data has been compiled to evaluate the carcinogenic potential of glyphosate. The expected normal biological variability for spontaneous tumor formation is reflected across this extensive data set (Tables 20–23). However, no specific neoplasm stands out as a consequence of glyphosate exposures. While some individual studies may note an increase in a specific neoplasm at the high dose, the pooled data fail to identify any consistent pattern of neoplasm formation, demonstrating that the effect is not reproducible and not treatment-related. The lack of a dose-response across the several orders of magnitude suggests that no individual tumor of single etiology is attributable to glyphosate administration.

Glyphosate has undergone repeated and extensive review by the United States Environmental Protection Agency (US EPA 1993), the European Union (EC 2002, Germany Rapporteur Member State 2015b) and the World Health Organization/Food and Agriculture Organization of the United Nations (WHO/FAO 2004b, WHO/FAO 2004a). With regard to potential carcinogenic effects of glyphosate, the unanimous outcome of these reviews has been that the data provide sufficient evidence to conclude that glyphosate should not be considered a carcinogen. Genotoxicity studies with glyphosate, conducted under conditions stipulated by internationally accepted testing guidelines and GLP, as reviewed in 2000 (Williams et al. 2000) and recently updated (Kier and Kirkland 2013), indicate that glyphosate clearly does not exhibit the properties of a DNA-reactive genotoxic carcinogen. This lack of mutagenicity rules out an important concern for carcinogenicity.

Mink et al. published a review of the available epidemiological studies that investigated possible associations between glyphosate and cancer diagnosed in humans (Mink et al. 2012). No evidence was found for a statistically significant positive association between cancer and exposure to glyphosate. While one Agricultural Health Study (AHS) publication mentions a “suggested association” between glyphosate use and multiple myeloma (De Roos et al. 2005), a later summary of AHS results note that there were no associations between glyphosate use and a number of cancers, including lymphohematopoietic cancers, leukemia, NHL, and multiple myeloma (Weichenthal et al. 2010). A subsequent reanalysis of AHS data obtained under the Freedom of Information Act notes no suggestion of an association between glyphosate use and multiple myeloma, with a relative risk of 1.1 and 95% and a confidence interval of 0.5–2.9 (Sorahan 2012). A recent review paper (Alavanja et al. 2013) cites another epidemiology study claiming an association between glyphosate use and NHL (Eriksson et al. 2008), but this research is strongly criticized in the recent Reevaluation Assessment Report for glyphosate Annex I Renewal in Europe (Germany Rapporteur Member State 2015b), highlighting potential referral bias, selection bias, uncontrolled confounding, limited data usage contrary to claims of including all new cases (living cases only, rather than living plus dead), and questionable definition/interpretation of dose-response. It is important to note that the Eriksson et al. study did detect statistically significant positive associations for small lymphocytic lymphoma/chronic lymphocytic leukemia and “unspecified NHL”, while the following lymphomas were not statistically significantly associated with glyphosate use: B-cell lymphomas, grade I-III follicular lymphoma, diffuse large B-cell lymphoma, other specified B-cell lymphomas, unspecified B cell lymphomas, and T-cell lymphomas (Eriksson et al. 2008). As previously discussed, statistically significant associations need to be evaluated further for study bias, confounders and sampling error, before expending resources and energy on further evaluation of potential causality.

Epidemiological investigations face the difficulty of reliably determining the magnitude of exposure to the chemical in question, while ruling out confounders like co-exposure to other chemicals, and environmental and lifestyle factors. In contrast, carcinogenicity studies in experimental animals, when conducted according to appropriate testing guidelines, are designed in a fashion that allows a direct association between observed effects and substance exposure, yet the relevance of observed findings to humans is an important consideration. This manuscript collectively presents the scientific community with carcinogenicity results from a remarkably large body of data from fourteen long-term carcinogenicity studies on glyphosate.

Glyphosate is of very low acute toxicity with an oral LD_50_ in the rat in excess of 5000 mg/kg of body weight.. The subchronic NOAEL is 400 mg/kg bw/day, and is based on effects that do not impair long-term survival (WHO/FAO 2004b, WHO/FAO 2004a). This allows administration of very high glyphosate doses to rodents for a prolonged time. Dietary levels of up to 30 000 and 40 000 milligrams of glyphosate per kilogram of diet have been administered to rats and mice, respectively, in chronic feeding studies covering their expected lifespan without apparent effects on longevity.

One of the most critical aspects of designing a carcinogenicity study is the choice of dose levels, especially the top dose, at either the limit dose or MTD. The relevant OECD TGs 451 and 453 for carcinogenicity studies propose a body weight depression of approximately 10% as evidence for systemic toxicity. This is equivalent to the concept of the MTD, which is discussed in a supporting OECD guidance document (OECD 2012b). For chemicals which are well tolerated by the experimental animal, where no dose-limiting toxicity is observed, the respective OECD guidance suggests 1000 mg/kg bw/day as the highest dose level (OECD 2012a). Many of the carcinogenicity studies performed in rats and mice with glyphosate have been conducted with the high dose group receiving levels of glyphosate at, or in excess of the limit dose because of its very low toxicity following repeat exposure. Following this extensive testing, even at very high exposure levels, there was no evidence of a carcinogenic effect related to glyphosate treatment. The select neoplasms highlighted in Tables 20–23 show normal biological background levels of spontaneous neoplasms, with lack of dose-response across the data sets. The combined studies clearly indicate that glyphosate's carcinogenic potential is extremely low or non-existent in animal models up to very high doses.

By way of comparison, the worst-case calculated human dietary exposure to glyphosate, the Theoretical Maximum Daily Intake (TMDI) is 0.14 mg/kg bw/day (EFSA 2012). Systemic exposure of operators, as assessed for the EU reapproval of glyphosate, is predicted to be between 0.0034 (German BBA model, tractor-mounted ground-boom sprayer) and 0.226 mg/kg bw/day (UK POEM, hand-held-spraying to low targets, data not shown). The model estimates are supported by human biomonitoring data in farmers showing systemic exposures of 0.004 and 0.0001 mg/kg/day for worst-case and mean acute doses, respectively (Acquavella et al. 2004). The high doses in chronic rodent studies at which no evidence of carcinogenicity is demonstrated are at least hundreds of thousands fold greater than peak human systemic exposure levels. Clearly, there is no scientific basis for concern of carcinogenic risk to humans resulting from glyphosate exposure.

With over 40 years of scientific research on glyphosate, no compelling evidence exists for a mechanism for glyphosate to cause cancer. Mammalian metabolism does not activate glyphosate to a toxic metabolite (Anadon et al. 2009, WHO/FAO 2004a). The lack of glyphosate DNA reactivity supports the lack of potential for an initiation event for carcinogenesis (Kier and Kirkland 2013). Clearly, there is a lack of potential for glyphosate to induce hormonal oncogenesis, based on both the tumor incidence data presented and the unequivocal evidence that glyphosate is not an endocrine disruptor (Bailey et al. 2013, Levine et al. 2012, Saltmiras and Tobia 2012, Webb et al. 2013, Williams et al. 2012).

The absence of test substance-related neoplastic findings in a total of 14 rodent cancer bioassays with glyphosate is in stark contrast to the recent dramatic media reports, internet postings, and YouTube videos of rat tumors, hypothesized to be caused by treatment with maize containing glyphosate residue or drinking water spiked with a glyphosate formulation (Seralini et al. 2014). Such reports, under the scrutiny of the global scientific community, demand greater data transparency and accountability within the peer review process.

**Figure 2.**
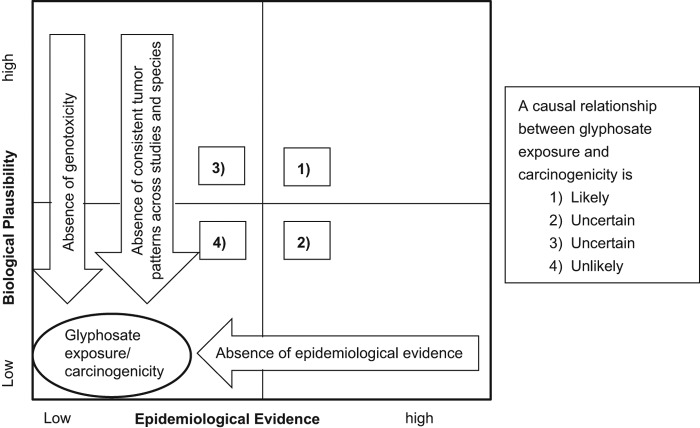
Likelihood of glyphosate carcinogenicity based on experimental and epidemiological data; a causal inference grid as proposed by Adami et al. (2011) to utilize both toxicological and epidemiological data.

The absence of a glyphosate-related mechanism for carcinogenesis, the huge volume of genotoxicity data studies indicating no likely mutagenic or DNA-reactive potential (Kier and Kirkland 2013), combined with the lack of epidemiological evidence for glyphosate-induced cancer (Mink et al. 2012), and the lack of carcinogenicity in multiple rodent carcinogenicity assays, are depicted in a causal inference grid in Figure 2, as put forth by Adami et al. (Adami et al. 2011). The overwhelming weight of the available evidence, demonstrating a lack of both biological plausibility and epidemiological effects, draws a compelling conclusion that glyphosate's carcinogenic potential is extremely low or non-existent.

## Supplementary material available online

Data Supplementary Study 1–14.
