# Supplementary material for: Evaluation of carcinogenic potential of the herbicide glyphosate, drawing on tumor incidence data from fourteen chronic/carcinogenicity rodent studies
Source: Crit Rev Toxicol. 2015 Feb 26;45(3):185–208. doi: 10.3109/10408444.2014.1003423 (PMC4819582; doi:10.3109/10408444.2014.1003423)
Supplement: Supplementary file 1 [file itxc_a_1003423_sm2515.zip › DataSupplementCombinedFilesUpdate_12-19-2014.pdf]

For the best experience, open this PDF portfolio in Acrobat 9 or Adobe Reader 9, or later.

Get Adobe Reader Now!
